# Booming Development of Group IV–VI Semiconductors: Fresh Blood of 2D Family

**DOI:** 10.1002/advs.201600177

**Published:** 2016-06-22

**Authors:** Xing Zhou, Qi Zhang, Lin Gan, Huiqiao Li, Jie Xiong, Tianyou Zhai

**Affiliations:** ^1^ State Key Laboratory of Material Processing and Die & Mould TechnologySchool of Materials Science and EngineeringHuazhong University of Science and Technology (HUST)Wuhan430074P. R. China; ^2^ State Key Laboratory of Electronic Thin Films and Integrated DevicesUniversity of Electronic Science and Technology of ChinaChengdu611731P. R. China

**Keywords:** 2D, electronics, group IV metal chalcogenides, optoelectronics, semiconductors

## Abstract

As an important component of 2D layered materials (2DLMs), the 2D group IV metal chalcogenides (GIVMCs) have drawn much attention recently due to their earth‐abundant, low‐cost, and environmentally friendly characteristics, thus catering well to the sustainable electronics and optoelectronics applications. In this instructive review, the booming research advancements of 2D GIVMCs in the last few years have been presented. First, the unique crystal and electronic structures are introduced, suggesting novel physical properties. Then the various methods adopted for synthesis of 2D GIVMCs are summarized such as mechanical exfoliation, solvothermal method, and vapor deposition. Furthermore, the review focuses on the applications in field effect transistors and photodetectors based on 2D GIVMCs, and extends to flexible devices. Additionally, the 2D GIVMCs based ternary alloys and heterostructures have also been presented, as well as the applications in electronics and optoelectronics. Finally, the conclusion and outlook have also been presented in the end of the review.

## 1 Introduction

2D layered materials (2DLMs) have ignited intensive attention since the discovery of graphene.^1^ These 2DLMs possess different properties such as electronic structures, large specific surface area, and the quantum confinement of electrons due to the ultrathin thickness compared with their bulk counterparts, thus paving a new way for the next generation electronic, optical, optoelectronic, and flexible systems.^2^, ^3^, ^4^, ^5^, ^6^, ^7^, ^8^, ^9^, ^10^, ^11^, ^12^, ^13^, ^14^, ^15^ Taking graphene, for example, the single layer of graphite has promising applications in broadband optical modulators^16^ and ultrafast high frequency photosensors^17^ due to the linear dispersion of the Dirac electrons.^18^ However, the absence of a bandgap has impeded the applications of graphene in nanoelectroncis. To overcome the disadvantages caused by the gapless band in graphene, intensive efforts have been strived, such as chemical doping, topography control, etc.^19^, ^20^, ^21^ Unfortunately, only very limited success has been achieved.^20^, ^22^, ^23^, ^24^ Consequently, uncovering other layered materials with bandgap is urgent. 2D layered metal chalcogenides (2DLMCs) is a class of graphene‐like structures with tight bonding along intralayer and weak van der Waals interactions between neighboring layers, making it possible for these materials to be exfoliated into single or few layers from bulk counterparts by mechanical or chemical methods. Unlike gapless graphene, these 2DLMCs have bandgaps in comparison to commercially silicon semiconductors, and the bandgaps can be further adjusted in a large range with thickness change. For example, it has been reported that MoS_2_ has emerged with a direct bandgap of 1.8 eV when reduced the thickness to monolayer.^25^ Because of this merit, Few layered 2DLMCs such as transition metal dichalcogenides (TMDs) (MoS_2_,^26^, ^27^, ^28^ WS_2_,^29^, ^30^, ^31^, ^32^, ^33^, ^34^, ^35^, ^36^ TiS_3_,^37^ etc.) and group III–VI nanomaterials (InSe,^38^, ^39^, ^40^, ^41^, ^42^ GaSe,^43^, ^44^, ^45^, ^46^, ^47^, ^48^ etc.) have been extensively investigated in many fields including photodetectors,^49^, ^50^, ^51^, ^52^, ^53^, ^54^, ^55^, ^56^, ^57^, ^58^ gas sensors,^59^, ^60^ field effect transistors (FETs),^38^, ^61^, ^62^, ^63^, ^64^, ^65^ and flexible devices.^66^, ^67^, ^68^

Among the 2DLMCs, group IV metal chalcogenides (GIVMCs, metal = Ge, Sn; chalcogen = S, Se, Te) provide an opportunity for sustainable electronic and photonic systems recently due to the low‐cost, earth‐abundant, and environmentally friendly features^69^ In 2013, ultrathin GIVMCs have been mechanically exfoliated for high performance FETs such as SnS_2_,^70^, ^71^, ^72^ SnSe_2_,^73^ SnS_2–x_Se_x_.^74^ In view of the difficulty in large‐scale synthesis via mechanical exfoliation, Peng and co‐workers^75^ in 2015 demonstrated for the first time the controlled synthesis of thin SnS_2_ nanosheets arrays by predefined metal seeds with the average thickness of 20 nm, thereby providing a new approach to the large‐scale production. Recently, Zhai and co‐workers^76^ first time achieved large area of ultrathin SnSe_2_ nanosheets (≈1.5 nm, corresponding to two layers) by employing a newly Sn precursor (SnI_2_), which further flourish the family of 2DLMCs. More importantly, 2D GIVMCs have demonstrated many interesting physical properties^13^, ^77^ and possible applications in electronics and photonics. Notably, tin sulfide (SnS) has been reported to be a native p‐type semiconductor due to the small enthalpy of formatting Sn vacancies, generating shallow acceptors.^78^ It has a high absorption coefficient (α > 10^4^ cm^−1^) across the direct absorption edge at 1.3–1.5 eV, rendering it a promising candidate for solar cells and photodetectors.^79^ Besides, SnSe_2_ has shown the advantage over the memory devices due to the significant changes of the optical reflectivity while heated by the laser and transformation from the amorphous to the crystalline state.^80^, ^81^, ^82^ Theoretical calculations predict that SnSe_2_ can transit from an indirect to direct bandgap semiconductor as the thickness decreased to single layer similar with MoS_2_, indicating the potential applications in optical and optoelectronic devices.^83^ In addition, GIVMCs have demonstrated excellent performance in FETs and photodetectors. For example, SnS_2_ has illustrated a relatively high carrier mobility up to 230 cm^2^ (V^−1^ s^−1^) and on–off current ratios over 10^6^,^72^ comparable or even surpassing many other 2DLMs.^84^, ^85^, ^86^, ^87^, ^88^ Ultrathin SnSe_2_ nanosheets have shown high responsivity of above 10^3^ A W^−1^ with a fast response time of ≈8 ms.^76^

In this review, we will present a comprehensive review for the research achievements in this field. The review mainly incorporates the intriguing physical properties (crystal structures, electronic structures), followed by the various methods for synthesis including mechanical exfoliation, solvothermal, and vapor deposition methods. Afterward, recent device developments of 2D devices in electronics and photonics such as FETs, photodetectors, and heterostructures based devices have been presented. Finally, the review is summarized with prospects for the future research in this area.

## 2 Crystal Structures

**Figure 1:**
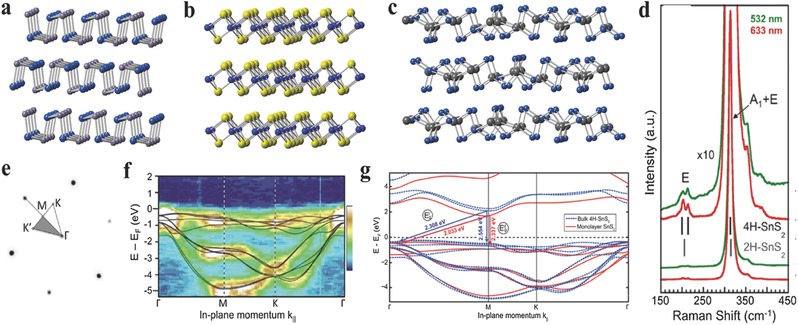
a–c) Crystal structures of layered orthorhombic, hexagonal, monoclinic phases of 2D GIVMCs. d) Raman spectra of bulk SnS_2_ at two excitation wavelengths (532, 633 nm). Gray and black vertical lines mark the main Raman lines of 2H–SnS_2_ and 4H–SnS_2_. e) Low‐energy electron diffraction pattern with overlaid irreducible wedge of the surface Brillouin zone. f) Angle resolved photoelectron spectroscopy of the projected band structure of bulk SnS_2_. False color scale: Dark blue, lowest intensity; white, highest intensity. Black lines are results of DFT band structure calculations for 4H–SnS_2_ using the HSE hybrid functional. g) Comparison of the calculated band structure of bulk (4H) and monolayer SnS_2_. (d–g) Reproduced with permission.^72^ 2014, American Chemical Society.

As an important group of layered materials, GIVMCs have strong covalent bonding existing in plane along the 2D direction while the weak van der Waals force dominating out of plane similar with other 2DLMs.^2^, ^4^ In general, GIVMCs have been classified into two groups according to their chemical compositions: MX (SiC,^89^ SiS,^90^ GeS,^91^, ^92^ GeSe,^93^, ^94^, ^95^, ^96^ SnS,^97^, ^98^, ^99^ SnSe,^100^, ^101^, ^102^, ^103^, ^104^, ^105^, ^106^, ^107^, ^108^ SnTe,^109^, ^110^) and MX_2_ (GeS_2_,^111^ GeSe_2_,^112^, ^113^, ^114^, ^115^ SnS_2_,^72^, ^116^, ^117^, ^118^ SnSe_2_ 73, 119, 120, 121, 122, 123, 124, 125). In this part, some typical GIVMCs are presented in **Table** **1**. The physical properties of GIVMCs are closely relative with their crystal structures, which paves a way for pursuing the basically issues in the application of electronics and optoelectroncis. Interestingly, GIVMCs have their unique characteristics existing in versatile crystal phases such as hexagonal and orthorhombic due to the different oxide states of the metals and chalcogen elements. For example, Sn‐chalcogenides could crystallize in orthorhombic space units to form a native p‐type SnS due to the small enthalpy of formatting Sn vacancies, generating shallow acceptors,^78^ in which Sn atoms with oxidation state of +2 are connected with three S ions to form the puckered Sn–S layers coupled by the weak van der Waals interaction with the space group of *Pnma* as shown in **Figure** **1**a.^69^, ^78^, ^79^, ^97^, ^98^, ^126^, ^127^, ^128^, ^129^, ^130^ Each single layer has a thickness of ≈0.56 nm. However, Sn‐chalcogenides can also crystallize into 2D hexagonal unit cells with the Sn oxidation state of +4, to form a native n‐type semiconductor, in which each single layer (≈0.59 nm) consists of one monolayer of Sn atoms sandwiched with between two layers of S atoms with the space group of *P‐*3*m*1 as illustrated in Figure 1b. In addition, all Ge‐chalcogenides (S, Se) are p‐type semiconductors, and the crystal structures of them belong to orthorhombic phase except GeSe_2_ revealing a monoclinic phase as shown in Figure 1c.^113^

**Table 1:** Summary of typical 2D GIVMCs and their properties. (Ind.: indirect; dir.: direct)

GIVMCs	Crystal structures	Lattice parameters[Å]	Distance[nm]	Electronic properties	Bulk bandgaps[eV]	References
GeS	Orthorhombic	a = 4.3	0.56	p‐type	ind./dir. 1.55–1.65	91, 92
		b = 10.47				
		c = 3.64				
GeS_2_	Orthorhombic	a = 11.74	–	p‐type	dir. 2.8–3.4	111, 260
		b = 22.47				
		c = 6.88				
GeSe	Orthorhombic	a = 10.84	–	p‐type	ind./dir. 1.1–1.2	93, 94
		b = 3.83				
		c = 4.39				
GeSe_2_	Monoclinic	a = 7.01	–	p‐type	2.8	113
		b = 16.79				
		c = 11.83				
SnS	Orthorhombic	a = 4.33	0.56	p‐type	ind. 1.07	79
		b = 11.19			dir. 1.3	
		c = 3.98				
SnS_2_	Hexagonal	a = b = 10.47	0.62	n‐type	ind. 2.2	72
		c = 5.89				
SnSe	Orthorhombic	a = 11.49	0.57	p‐type	ind. 0.9	105
		b = 4.15			dir. 1.3	
		c = 4.44				
SnSe_2_	Hexagonal	a = b = 3.81	0.62	n‐type	1.0	120
		c = 6.14				

Raman spectroscopy is a useful instrument to verify the crystal phase and make a distinction between bulk and few or mono layered counterparts of 2DLMs.^71^, ^72^, ^131^ Generally, metal chalcogenides could crystallize to the following phases such as 2H, 4H, and 1T structures. 2H and 4H phases are semiconducting while 1T phase is metallic. 2H phase has been investigated extensively for electronics and optoelectronics. These MX_2_ can exhibit different polytypes in the same structure of X–M–X layers but different interlayer stacking. Taking SnS_2_, for example, 2H–SnS_2_ belongs to space group $D_{3d}^{3}$ (*P‐*3*m*1) and has three in the unit cell which extends over one sandwich layer, while 4H–SnS_2_ belongs to space group $C_{6v}^{4}$ (*P*6_3_ *mc*) and includes six atoms in the unit cell, which extends over to two sandwich layers.^132^ For 4H–SnS_2_ polytype, the most intense Raman peak emerging at 313.5 cm^−1^ can be ascribed to a mixture of A_1_ and E optical modes, while the E‐mode results in a doublet at 200 and 214 cm^−1^ as shown in Figure 1d.^72^ However, in 2H–SnS_2_ polytype, the stronger peak at 315 cm^−1^ comes from A_1g_ mode, while the E_g_ mode gives rise to a single peak at 205 cm^−1^.^118^ In addition, the relation between intensities of the Raman peaks and the thickness of the layered SnS_2_ crystals has also been investigated. In general, the E_g_ Peak would become weak even undetectable due to the reduction of the scattering centers for in‐plane scattering, as the thickness decreases down to nanoscale.^118^, ^130^ What is more, Sutter and co‐workers^72^ have also explored the Raman scattering from the most intense (A_1_ + E) optical phonon mode in few‐layered 4H–SnS_2_ polytype with the zone‐center optical phonon mode of Si at ≈520 cm^−1^ serving as a reference. Consequently, as the thickness of the 2D SnS_2_ crystal decreased down to monolayer or bilayer which is below that of the single unit cell of the 4H‐polytype, the (A_1_ + E) mode transits into A_1g_ mode which reveals a low intensity. The relationship between intensity ratio of I(SnS_2_)/I(Si) and the thickness of the layered crystals has also been plotted. Over the range from monolayer to 20 layers, the I(SnS_2_)/I(Si) ratio increases closely linearly with the number of layers.

Electronic structures are elementary for exploring the electronic and optical processes in versatile electrical and optical devices. The electronic band diagram of graphene shows a linear energy dispersion at the K point, thus resulting in a gapless band structure.^1^, ^20^, ^133^, ^134^, ^135^, ^136^, ^137^, ^138^, ^139^ Consequently, the gapless band structure of graphene results in the low controllability of electronics and inferior photoresponsivity,^2^, ^5^, ^27^, ^134^ which impedes the applications in electronics and optoelectronics. The electronic structures of typical GIVMCs are summarized in Table 1. The bandgaps of the GIVMCs varies in a wide range from ≈1 eV ≈3.4 eV, and are calculated to have indirect and direct bandgaps in the bulk counterparts. Taking SnS_2_, for example, Sutter and co‐workers^72^ have employed micro‐angle‐resolved photoelectron spectroscopy (micro‐ARPES) band mapping to study the electronic structure on micrometer sized exfoliated 4H–SnS_2_ crystal. As shown in Figure 1e, the low‐energy electron diffraction (LEED) pattern obtained at a *E* = 28 eV from the exfoliated samples reveal sharp spots with hexagonal symmetry as expected, and also exhibits two sets of first‐order diffraction spots with interactive low and high intensity, which verifies the interlayer stacking of the 4H–SnS_2_ polytype. They also obtained high‐quality micro‐ARPES band structure maps with an energy resolution better than 300 meV at room temperature, employing energy‐filtered photoelectron angular distributions mapped in reciprocal space as demonstrated in Figure 1f. Furthermore, the electronic band structure of bulk and monolayer SnS_2_ crystals employing ab initio density‐functional theory (DFT). The calculations show that as transiting from bulk to monolayer SnS_2_ the energy of the valence band maximum (along (Γ‐M)) remains approximately the same. The conduction band minimum at M for the monolayer structure undergoes a downward shift of 245 meV compared to the bulk (Figure 1g). This transition is different from MoS_2_ of which the transition to a direct bandgap resulting from a significant shift of the valence band edge at Γ.^72^ However, it is worth noting that the bandgap is only weakly affected and remains indirect in the transition from bulk to monolayer SnS_2_.

## 3 Preparation Methods and Characterizations

Up to date, reliable production of ultrathin 2D GIVMCs is primarily for further research and applications. Generally, the preparation methods can be classified into top‐down and bottom‐up methods, such as mechanical exfoliation,^72^, ^92^, ^116^ solvothermal method,^97^, ^129^, ^140^, ^141^ vapor deposition,^69^, ^76^, ^118^, ^142^ atomic layer deposition,^126^, ^143^ and so on. In the following context, we will focus on four methods.

### 3.1 Mechanical Exfoliation

**Figure 2:**
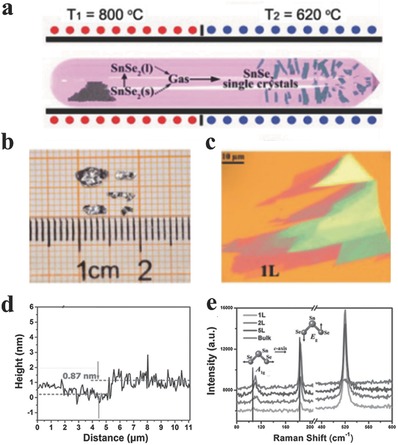
a) The schematic illustration of the growth of SnSe_2_ single crystals using the vapor transport deposition (VTD) technique. b) The optical image of SnSe_2_ single crystals with the size of several millimeters. c) Optical image of SnSe_2_ flake placed on the surface of a silicon wafer capped by 285 nm thick silicon dioxide. d) The thickness of monolayer SnSe_2_. e) Normalized Raman spectra of SnSe_2_ from monolayer to bulk. The intensity of Si peak is set as constant. (a–e) Reproduced with permission.^120^

Mechanical exfoliation method has been widely employed for obtaining few‐layer or monolayer nanoflakes from their bulk counterparts since its initial utilization in fabricating single layer graphite by Novoselov et al.^[1]^ Generally, mechanical exfoliated flakes possess higher quality and clean surface states on a variety of substrates, appropriate for fundamental physics studies and advanced devices.^10^, ^27^ Up to now, most studied ultrathin 2D GIVMCs have been fabricated by mechanical cleavage from the high‐quality single crystals that were obtained via the chemical vapor transport (CVT) method as illustrated in **Figure** **2**a. The raw materials were first sealed in a long quartz tube under very severe vacuum condition such as 10^−6^ Torr. Then the quartz tube is placed in a two‐zone furnace with the reaction zone a higher temperature while the collection zone at a lower temperature for several days or even several months.^92^, ^120^ The shining bulk single crystals have been obtained after the reaction in Figure 2b. For obtaining few‐layer or monolayer materials, bulk single crystals would be pressed against an adhesive scotch tape and consequent tautological cleavage gives rise to the flakes pasted on to the tape, which can be transferred onto a variety of substrates as shown in Figure 2c. Few‐layer or monolayer could usually be found among these thicker flakes, with the thickness confirmed by atomic force microscopy (AFM) profile measurements (Figure 2d) and the crystal phase identified by the Raman spectroscopy (Figure 2e).^72^, ^73^, ^92^, ^120^, ^121^

Although mechanical cleavage method is fairly easy to obtain high quality nanoflakes, the extremely low yield and the low controllability of the layer number and large‐area uniformity have harshly restricted it from practical applications in electronics and optoelectronics. On the contrary, bottom‐up methods have been widely researched for large scale throughout, which we will present in the following sections.

### 3.2 Solvothermal Method

**Figure 3:**
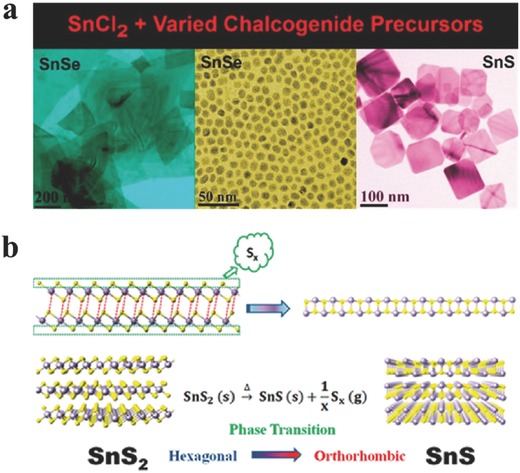
a) New methods for synthesizing SnE (E = S, Se) with different morphologies. Reproduced with permission.^129^ 2014, American Chemical Society. b) Schematic diagram illustrating the phase transition process between SnS_2_ and SnS. After annealing, SnS_2_ dissociation occurs, and S depletion and high temperatures promote dissociation processes. Reproduced with permission.^117^ 2014, American Chemical Society.

The solvothermal methods allow one to get a relatively large quantity of products, which can solve the problems of low yield of the products via mechanical cleavage methods. Most employed solvothermal methods for synthesizing GIVMCs can summarized by the following procedure: Sn‐halogenides (Cl used mostly) + varied chalcogenide precursors, as shown in **Figure** **3**a.^97^, ^117^, ^119^, ^140^, ^141^, ^144^, ^145^, ^146^, ^147^ Usually the selection of chalcogenides and the combination of ligands can play crucial roles in determining the morphology, sizes, yield and crystal phases of the as‐synthesis products. Consequently, the as‐synthesized products are employed to fabricate metal–semiconductor–metal (MSM) device to study the optoelectronic properties and demonstrate the potential use in optoelectronics and photovoltaics. Interestingly, Guo and co‐workers^117^ have performed the subsequent structural phase transition process in argon from SnS_2_ to SnS structures. As demonstrated in Figure 3b, the formation of this phase transition may depend on the S depletion in the annealed process. First, SnS_2_ dissociation occurs in the high S availability regime. Then, dissociation process is promoted by the S depletion and high temperatures. This route is on the basis of the inconsistent sublimation of SnS_2_, leading to a direct transition from SnS_2_ to SnS.^117^, ^148^ What is more, this route paves a new way to alter the properties of a product without any additional elements through structural phase transition, which may enlighten the applications in electronics and optoelectroncis.

In spite of the advantages over the relatively high quantity of as‐synthesized materials via solvothermal methods, the resulting products are usually contaminated with excessive impurity doping or adhered by the ligands used in the processes, which would impact the performance of electronics and optoelectronics. In contrast, vapor deposition methods, both chemical vapor deposition (CVD) as with physical vapor deposition (PVD), have been probed extensively for large‐area uniform, high quality, and morphology controllable products recently.

### 3.3 Vapor Phase Deposition

The vapor phase deposition process is widely employed for atomic‐scale control of 2D materials, particularly semiconductors. The growth procedure via vapor deposition involves either single solid precursor of destination product, or codeposition of sulfur or selenium and solid precursor onto various substrates such as SiO_2_/Si, and mica.

#### 3.3.1 Physical Vapor Deposition

**Figure 4:**
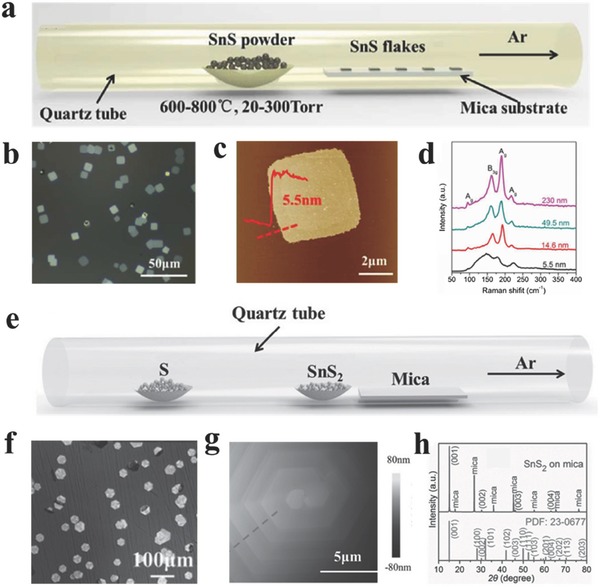
a) Schematic illustration for PVD growth of 2D SnS flakes. b) Optical image of the 2D SnS flakes. c) AFM image and height information of one 2D SnS flake. d) Raman spectra of the SnS flakes with different thickness. (a–d) Reproduced with permission.^79^ 2016, The Royal Society of Chemistry. e) Schematic illustration for synthesizing 2D SnS_2_ crystals. f) Optical image of large scale 2D SnS_2_ crystals. g) AFM image of the center region of a thick crystal showing hexagonal spiral fringes. h) XRD pattern of the SnS_2_ sample. (e–h) Reproduced with permission.^149^

There have been some attempts to synthesize 2D GIVMCs materials via simple evaporization of bought powders such as SnS,^69^, ^79^ SnSe,^105^ and SnS_2_.^149^ Meng and co‐workers^79^ employed SnS powder as the only source for evaporation. Newly cleaved mica sheets were placed downstream of ≈8–20 cm away from the center zone to collect the products. The reaction was performed at a temperature of 600–800 °C with the chamber pressure around 20–300 Torr under argon flow (**Figure** **4**a). Large scale of 2D ultrathin SnS nanoflakes were obtained via this simple PVD method as shown in Figure 4b. the typical thickness is identified to be about 14.6 nm. The thickness can be found to be as thin as 5.5 nm corresponding to ≈10 layers confirmed by the AFM profile in Figure 4c, and the thinner nanosheets have rough edges and surface, partially resulting from imperfect growth at lower temperature. Then the vibrational properties of SnS nanoflakes with different thickness have been researched via Raman characterization as shown in Figure 4d. For thicker SnS flakes, there exists four clearly observed Raman peaks at 95.5, 190.7, and 216.8 cm^−1^ assigned to A_1g_ modes, whereas the peak at 162.5 cm^−1^ associated with B_3g_ mode. However, the Raman peaks become seriously deviation and undetectable as the thickness decreases down to 5.5 nm, which may result from the inferior crystallinity of the as‐synthesized SnS flakes at lower temperature. Furthermore, polarized Raman characterization was implemented to explore the polarization dependence due to the reduced symmetry of the orthorhombic phase. The authors inspected that the orthorhombic phase SnS nanosheets exhibit strong anisotropic Raman response similar with black phosphorous^150^ and ReS_2_.^151^, ^152^, ^153^, ^154^ The parallel‐polarization configuration strongly suggests that the A_1g_ mode (190.7 cm^−1^) can be employed to detect crystallographic orientation of the SnS flakes because of the A_1g_ mode reaches the maximum as illumination light polarization is parallel to armchair direction of the SnS flakes, which may also exist in other group IV orthorhombic phases such as SnSe, GeS, and GeSe. Later, Meng and co‐workers^149^ used SnS_2_ powder as the evaporation source at the center zone under ambient pressure (Figure 4e). S powder was also adopted to avoid the transition of SnS_2_ to SnS phase. Consequently, large‐scale of SnS_2_ nanoflakes were obtained with typically lateral sizes ranging from 13 to 43 μm (Figure 4f). X‐ray diffraction (XRD) characterization in Figure 4h indicates the as‐synthesized SnS_2_ crystals have a 2T‐type hexagonal phase structure with lattice constants of a = b = 0.365 nm, c = 0.589 nm (JCPDS PDF card: 23–0677). Interestingly, the AFM profile of the nanoflake in Figure 4g reveals clear dislocation hillock consisting of several spiral patterns on the surface, suggesting that the SnS_2_ flakes follow a screw dislocation driven (SDD) spiral growth mode.

#### 3.3.2 Chemical Vapor Deposition

**Figure 5:**
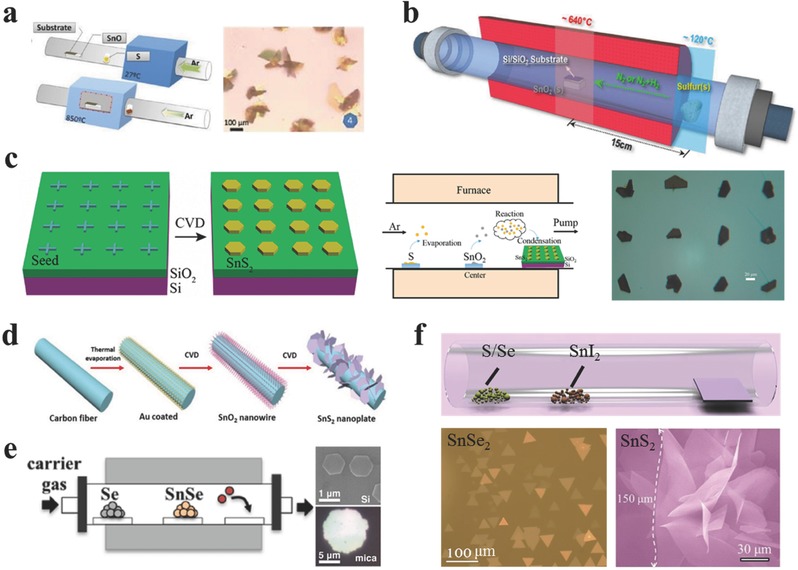
a) Illustration of the growth process and optical image of the as‐prepared SnS_2_ flakes. Reproduced with permission.^186^ 2016, The Royal Society of Chemistry. b) The vapor transport set‐up for polymorphic growth of the SnS_2_ and SnS crystals. Reproduced with permission.^130^ 2015, American Chemical Society. c) Schematic diagrams of patterning seed arrays and the CVD experimental setup, and optical image of the as‐synthesized SnS_2_ crystal arrays. Reproduced with permission.^75^ 2015, American Chemical Society. d) Schematic diagram of experimental setup. Reproduced with permission.^142^ 2015, The Royal Society of Chemistry. e) Schematic illustration of the synthetic setup, SEM, and optical images of the as‐synthesized SnSe_2_ nanoplates. Reproduced with permission.^188^ 2013, American Chemical Society. f) Schematic diagram of the CVD synthetic process, optical image of large‐scale SnSe_2_ nanoflakes (Reproduced with permission.^76^) and SEM image of large‐size SnS_2_ nanosheets. Reproduced with permission.^118^

CVD methods have been attempted intensively due to the obtained high quality, large‐area, and uniform products. CVD‐grown graphene has made great breakthrough in the growth of large‐area graphene.^17^, ^155^, ^156^, ^157^, ^158^, ^159^, ^160^ Recently, the synthesis of 2DLMs via CVD methods has been illustrated in many reports especially for MoS_2_,^30^, ^31^, ^161^, ^162^, ^163^, ^164^, ^165^, ^166^, ^167^, ^168^, ^169^, ^170^, ^171^, ^172^, ^173^, ^174^, ^175^, ^176^, ^177^, ^178^, ^179^, ^180^, ^181^, ^182^, ^183^, ^184^, ^185^ which also shows promising applications in electronics and optoelectronics. Up to now, there are several reports on synthesizing 2D GIVMCs nanoflakes via CVD method, which is still at the initial stage. The growth process 2D GIVMCs nanoflakes is performed via thermal evaporation of different precursors and using various substrates to collect products, under an inert gas (e.g., Ar) protection, or as with hydrogen. Among so many solid precursors, metal oxides are the most widely employed. Li and co‐workers^186^ proposed that SnS_2_ is easy to resolve into Sn and S at 650 °C impeding the synthesis of high quality materials at higher temperature. Therefore, they employed fast heating and cooling process with the sulfurization of SnO powder at high temperature of 850 °C as shown in **Figure** **5**a. They obtained thick SnS_2_ flakes with hexagonal and half hexagonal shapes, randomly dispersing on the SiO_2_/Si substrate with the thickness up to 100 nm. Then they performed a series of growth processes with different reaction time from 1 to 4 min, and draw the following conclusions: first, SnO powders were evaporated and deposited on the substrate. Then, SnO particles on the substrate initiated reacting with S resulting in the formation of small SnS_2_ particles. Finally, SnS_2_ grew larger based on the nuclei. They further inspected that the (001) plane has higher reticular densities, larger interlayer distances and lower surface energy than other planes,^187^ so the atoms in this plane are easier to adsorb other different atoms according to the Bravais rule. However, the SnS_2_ nuclei were irregular and randomly dispersed on the substrate with various directions at the initiate stage, which may partially explain the growth mechanism.

Jo and co‐workers^130^ calculated free energy changes of formation of SnS_2_ and SnS from SnO_2_ and S, and concluded that the change in Gibbs free energy for SnS growth with H_2_ addition is negative and is lower than that of SnS_2_ in the same temperature. Therefore, they synthesized 2D polymorphous crystals of Sn‐sulfides on SiO_2_/Si substrates via sulfurization of SnO_2_ powder in a 12 in. length hot wall quartz tube furnace (Figure 5b). SnO_2_ powder was put into a porcelain boat at the center zone. The SiO_2_/Si substrate was mounted on the top of the boat with upside down and the center temperature was gradually increased to 620–680 °C, keeping for 15 min for the growth under the vacuum of 700–800 Torr. The mixture–gas flow ratio of nitrogen and hydrogen is vital to determine the crystal morphology with orthorhombic SnS or hexagonal SnS_2_. Typically, they found that 2D SnS nanosheets were stabilized for the H_2_/N_2_ ratio was larger than 0.4 with increasing the thickness with the flow ratio. Below the ratio of 0.4, the as‐synthesized products began to form irregular shapes, suggesting a transition from orthorhombic SnS to hexagonal SnS_2_. Consequently, when H_2_ was absence_,_ the hexagonal SnS_2_ crystals were formed stabilized with triangular shapes. This controlled synthesis of polymorphic 2D tin‐sulfides of either p‐type SnS or n‐type SnS_2_ also paves a new way for fabricate p‐n heterostructures for rectifiers and photovoltaic cells.

Peng and co‐workers^75^ reported for the first time controlled CVD synthesized 2D SnS_2_ nanosheets arrays at predefined locations on SiO_2_/Si substrates. First, they pattern nucleation sites (Pd/Cr, or Ni arrays) on SiO_2_/Si via nanofabrication routes, and cross marks were shown in Figure 5c. These substrates with metal arrays were used as the seed to collect products. In a typical CVD process, sulfurization of SnO_2_ powder was performed at 710 °C for 5 min under a vacuum of 30 kPa with an argon flow of 60 sccm. Finally, the patterned 2D SnS_2_ nanosheets with average thickness of 20 nm were synthesized.

Furthermore, He and co‐workers^142^ synthesized large scale half‐hexagonal nanosheets on carbon cloth through a two‐step method employing SnO_2_ nanowires as the template for growing SnS_2_ nanosheets. As illustrated in Figure 5d, they first grown large scale SnO_2_ nanowire arrays on carbon cloth, and then synthesized SnS_2_ nanosheets through sulfurization of these SnO_2_ nanowires with the lateral sizes limited to several microns and the thickness of tens of nanometers. They provided a method for growing 2D crystals under new conditions.

SnSe has also been explored to synthesize SnSe_2_ nanoflakes. For example, Cao and co‐workers^188^ synthesized SnSe_2_ nanoplates via a simple selenylation of SnSe powder and demonstrated that the growth procedure is intensively influenced by substrates. In a typical growth, ≈25 mg SnSe powder and ≈45 mg Se powder were put into two separated quartz boats, with the SnSe powder located at the center zone while Se powder at the entry of the furnace (Figure 5e). SiO_2_/Si and freshly cleaved mica as substrates were employed to collect the products. The center temperature was heated to 550 °C, and the Se at 350 °C with 10–30 sccm argon and 20–40 Torr in total pressure. Though the thickness is similar with ≈60 nm on both substrates, the lateral sizes of the nanoplates grown on mica were obviously larger than those grown on SiO_2_/Si (Figure 5e). They concluded that the observed morphological difference may be related to the different migration energy of SnSe_2_ adatoms on the substrates, which includes on‐substrate migration M1 and on‐nanoplate migration M2. Mica or SnSe_2_ has a clean surface completely passivated with no dangling bonds resulting in lower migration energy, compared with silicon, which has dangling bonds at the surface. Therefore, the migration rate on mica was higher than that on the nanoplate, M1_mica_ ≥ M2, resulting in the larger diameter of the nanoplates on the mica. It may pave the way for the controllable synthesis of other 2DLMCs. Lately, He and co‐workers^122^ synthesized 2D SnSe_2_ and SnSe nanosheets collected at lower and higher temperatures respectively through a one‐step CVD method. Just like the above method, they employed Se and SnSe powders as the precursors separated at upstream and center, respectively. A relative high reaction temperature of 750 °C was adopted. Accordingly, atoms were inclined to desorb into the carrier gas on substrates of higher temperatures. Therefore, the adsorbed Se atoms tended to rapidly desorb into the environment at higher temperatures, resulting in the formation of orthorhombic SnSe at higher temperatures and hexagonal SnSe_2_ at lower temperatures.

Recently, Zhai and co‐workers^76^ adopted a newly Sn precursor (SnI_2_) and achieved large area of SnSe_2_ nanosheets with ultrathin thickness. They considered that SnI_2_ takes some advantages over other solid precursors such as low melting point (≈320 °C), which may providing more uniform and stable growth conditions during CVD procedure for the growth of ultrathin SnSe_2_ flakes. What is more, for tin, iodine (coming from the reaction: SnI_2_ + 2Se = SnSe_2_ + I_2_) had been proposed to be an optimal carrier agent, since it was employed for growing copper–zinc–tin–sulfide (CTZS) single crystals with success. The typical CVD experiments were demonstrated in Figure 5f. 10 mg SnI_2_ powder and 100 mg Se powder were put into two separated porcelain boats. Freshly cleaved mica sheets were located at the downstream to collect the synthetic products. The reaction temperature was at 600 °C for 15 min and a mixture of argon (20 sccm) and hydrogen (5 sccm) was used at ambient atmosphere. After that, large‐area ultrathin SnSe_2_ flakes with triangular shapes were obtained illustrated in Figure 5f. Zhai and co‐workers for the first time synthesized high‐quality ultrathin few‐layered single‐crystalline SnSe_2_ flakes through this improved CVD method, and the photodetectors based on these high‐quality flakes showed exciting performance, including a high responsivity (1.1 × 10^3^ A W^−1^), a fast response time of 8.1 ms, superior to most reported 2DLMCs based photodetectors. Lately, Zhai and co‐workers^118^ further extended this improved CVD method to large‐size growth of ultrathin SnS_2_ nanosheets. In this process, sulfide took the place of selenium and SiO_2_/Si was employed as the substrate. Therefore, large‐size SnS_2_ nanosheets with the side length over 150 μm were obtained. This improved CVD method has shown its advantages over synthesis of high‐quality large‐area ultrathin 2D materials, which may extend to the synthesis of other 2DLMCs.

### 3.4 Ternary Alloys

**Figure 6:**
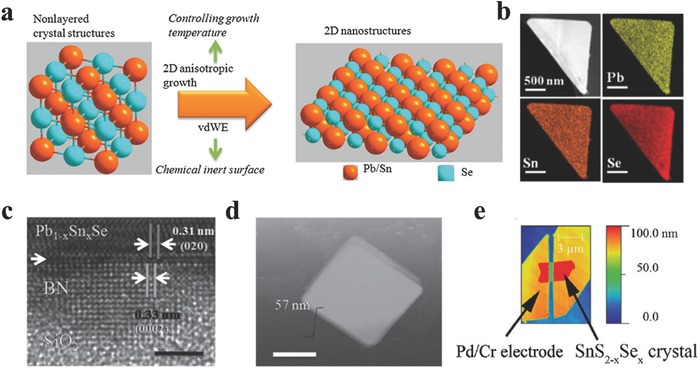
a) Schematic illustrations of van der Waals epitaxial ultrathin 2D Pb_1–x_Sn_x_Se nanoplates with a cubic crystal structure tailoring into ultrathin 2D nanostructures at certain growth temperature. b) TEM‐EDX mapping of triangle Pb_1–x_Sn_x_Se nanoplate. c) High‐resolution TEM of few‐layer BN/Pb_1–x_Sn_x_Se nanoplate heterostructure which denotes a clear interface between BN and Pb_1–x_Sn_x_Se. The red arrow shows the interface. The lattice mismatch between (020) surface of Pb_1–x_Sn_x_Se and (0002) surface of BN reaches 6.1%. Scale bar = 3 nm. d) AFM image of a typical Pb_1–x_Sn_x_Se nanoplate. Scale bar = 2 μm. e) An AFM image of a SnS_2–x_Se_x_ device with x = 0.4. (a,b) Reproduced with permission.^104^ 2015, American Chemical Society.(c,d) Reproduced with permission.^107^ (e) Reproduced with permission.^74^ 2013, American Institute of Physics.

Ternary alloys have attracted intensive attention in recent years due to the varied properties through doping the third elements into the pure binary systems, which may provide an important versatility in low‐power consumption electronics and optoelectroncis.^189^, ^190^, ^191^, ^192^, ^193^, ^194^ For example, Pb_1–x_Sn_x_Se is a narrow direct bandgap semiconductor with promising applications in mid‐infrared photodetection (1–3 μm), topological crystalline insulators and high‐speed logic devices due to the doping of Pb.^104^, ^107^, ^195^, ^196^ He and co‐workers^104^ reported the synthesis of ultrathin Pb_1–x_Sn_x_Se nanoplates via a simple doping process. The mixture of Pb and SnSe powders were used as the source placed at the center zone of 550–650 °C, mica and SiO_2_/Si were located at the downstream to collect products (**Figure** **6**a), confirmed by the transmission electron microscope (TEM)–energy dispersive X‐ray (EDX) mapping of a triangle Pb_1–x_Sn_x_Se nanoplate as shown in Figure 6b. The ultrathin Pb_1–x_Sn_x_Se nanoplates were obtained and showed high performance for mid‐infrared photodetectors. Furthermore, they directly grown ultrathin Pb_1–x_Sn_x_Se nanoplates on BN of which the surface is free of dangling bonds allowing the direct growth of a highly lattice‐mismatched heterostructures, characterized by the high‐resolution TEM image and AFM profile of the heterostruture (Figure 6c,d). Therefore, the relaxed strain and less surface states at the interface of epilayer and BN can not only improve their electronic properties but also promote their applications in integration techniques.^107^ In addition, the bandgap of selenium doped SnS_2_ can be tuned from 2.1 eV (SnS_2_) to 1.0 eV (SnSe_2_) through changing the Se content. What is more, this ternary alloys have been reported by Peng and co‐workers^74^ and Chen and co‐workers^197^ via a simple CVT method, and demonstrated promising applications in FETs and phototransistors (Figure 6e).

### 3.5 Heterostructures Based on 2D GIVMCs

**Figure 7:**
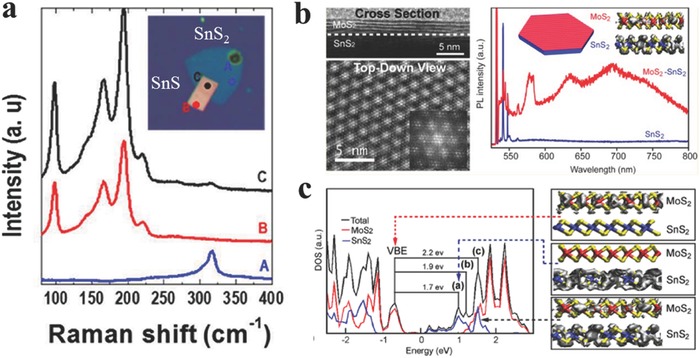
a) Raman spectra of SnS_2_ (blue), SnS (red), and the overlap region (black). The inset shows the corresponding optical image of vertical heterostructure. Reproduced with permission.^130^ 2015, American Chemical Society. b) Cross‐sectional TEM image and PL spectra of the MoS_2_/SnS_2_ heterostuctures. c) Calculated total and projected density of state for the 5‐L MoS_2_/SnS_2_ heterostructure, and charge density for several states of the 1‐L MoS_2_/SnS_2_ heterostructure: the valence band edge (VBE) top, a representative state within peak (a) (middle), and a mixed‐character state within peak (c), lying approximately 1.8 eV above the Fermi level (bottom). (b,c) Reproduced with permission.^245^ 2014, American Chemical Society.

Van der Waals (vdW) heterostructures composed of 2D layered materials have been attempted intensively recently due to the novel physical properties covering a wide range of electronic, optical, and optoelectronic systems.^198^, ^199^, ^200^, ^201^, ^202^, ^203^, ^204^, ^205^, ^206^, ^207^, ^208^, ^209^, ^210^, ^211^, ^212^, ^213^, ^214^, ^215^, ^216^, ^217^, ^218^, ^219^, ^220^, ^221^, ^222^, ^223^, ^224^, ^225^, ^226^, ^227^, ^228^, ^229^, ^230^, ^231^, ^232^, ^233^, ^234^, ^235^, ^236^, ^237^, ^238^, ^239^, ^240^, ^241^, ^242^ Jo and co‐workers^130^ synthesized polymorphic 2D tin‐sulfides of either p‐type SnS or n‐type SnS_2_ via adjusting hydrogen during the process. Then they fabricated p‐n heterostructures based on synthesized SnS and SnS_2_ as shown in **Figure** **7**a. Lately, Xing and co‐workers^243^ for the first time demonstrated a room temperature Esaki tunnel diodes based on exfoliated black phosphorous (BP) and tin diselenide (SnSe_2_) which possess a broken‐gap energy band offset. The device construction began with the cleavage of BP flakes onto a 285 nm SiO_2_/Si substrate via scotch tape. SnSe_2_ flake as the second layer was first adhered to commercially available elastic films as stamps supplied by Gel‐Pak, and then was aligned with the target BP flake under an optical microscope with micromanipulators. Javey and co‐workers^244^ further fabricated 2D–2D tunneling FETs based on exfoliated WSe_2_ and SnSe_2_ heterostructures employing ZrO_2_ as the gate dielectric, allowing the scaling of gate oxide to improve the subthreshold swing of the device. They presented high performance 2D–2D tunneling FETs such as the subthreshold swing of 100 meV and the maximum switching ratio *I*_on_/*I*_off_ of 10^7^.

However, the heterostructures fabricated by mechanical transfer procedure may remain great challenge such as the reproducibility, accuracy, and nondestructive transfer of the 2D flakes. Especially, the dangling bonds on the interface of the heterostructures by mechanical transfer procedure may adsorb other molecules such as oxygen or water, resulting in passivation which is harmful for advanced electronic devices. Therefore, there are many attempts on the epitaxial growth of heterostructures.^245^, ^246^ Jin and co‐workers^245^ have achieved heteroepitaxial growth of thin layers of MoS_2_, WS_2_, and WSe_2_ on SnS_2_ microplates via a CVD procedure employing metal chlorides and sulfide or selenium powders at low temperature (<500 °C). Taking MoS_2_/SnS_2_ heterostructure, for example, typical PL spectra of a SnS_2_ microplate and MoS_2_/SnS_2_ microplate were demonstrated in Figure 7b. Those sharp peaks from 540–562 nm of both spectra were assigned as the Raman peaks of Si, SnS_2_ and MoS_2_. For MoS_2_/SnS_2_ microplate, the peaks at 631 nm (1.97 eV) and 690 nm (1.80 eV) were slightly red shift compared with monolayer MoS_2_, suggesting the existence of few‐layered MoS_2_. Interestingly, there existed a peak at 578 nm (2.15 eV), which has never been observed in any MoS_2_ flakes. They further carried out density functional theory (DFT) calculations using the Vienna ab initio simulation package (VASP) for understanding the observed optical properties (Figure 7c). Excitation from the valence band edge (VBE) to a delocalized state with peak (c) in Figure 7c, was more likely to exist due to enhanced overlap. Photoluminescence spectrum from the delocalized state down to the VBE was calculated to be 2.2 eV, consistent with the newly existed peak at 578 nm (2.15 eV). Their calculations suggested that the newly observed peak at 578 nm mainly results from the coupling between MoS_2_ and SnS_2_.

## 4 Device Applications

The electronic band graph of graphene shows linear energy dispersion at the K point resulting in a gapless band structure. The gapless band structure of graphene causes a low controllability of electronics and inferior photoresponsivity, which impedes the applications in electronics and optoelectronics. However, 2D GIVMCs have a large bandgap range from 0.9 to 3.1 eV and the low‐cost, earth‐abundant, and environmentally friendly features render 2D GIVMCs particularly desirable for next‐generation electronics and optoelectronics. The following context will present an overview of the advances in electronics and optoelectronics based on 2D GIVMCs.

### 4.1 FETs Based on 2D GIVMCs

**Figure 8:**
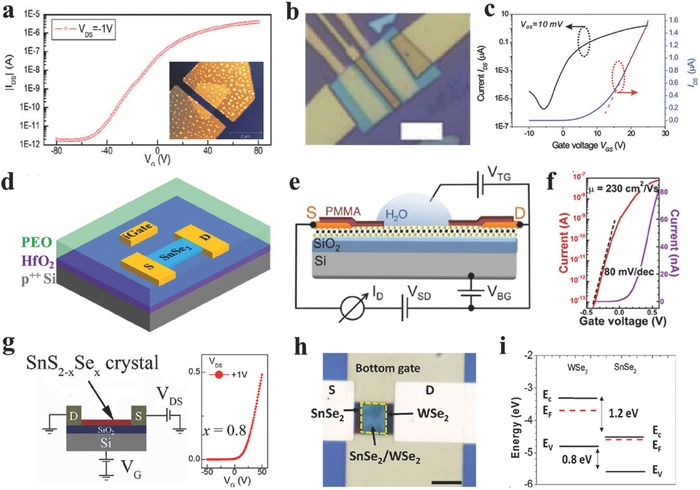
a) The drain–source current *I*_ds_ as a function of the gate voltage varied from −80 to 80 V. The inset shows the AFM image of the device. Reproduced with permission.^70^ 2013, Institute of Physics. b) Optical image of the top‐gated device, scale bar = 5 μm. c) Transfer curves of the studied top‐gated FET, within twenty voltage gated range, the *I*_ds_ can be tuned in seventh‐order range. The red dotted line is the linear fit to *I*_ds_ *–V*_g_ for *V*_th_. (b,c) Reproduced with permission.^71^ 2013, The Royal Society of Chemistry. d) Schematic diagram of the geometry of SnSe_2_ transistor covered with polymer electrolyte using 70 nm HfO_2_/p^++^ Si as back gate. Reproduced with permission.^121^ 2016, American Institute of Physics. e) Schematic diagram of the geometry of the SnS_2_ FET devices with SiO_2_/Si back gate and H_2_O solution top gate. f) Analogous transfer characteristics of a deionized water top‐gated SnS_2_ device, measured with dual top gate voltage sweeps, starting at +0.5 V, with turnaround points between +0.2 and −0.4 V. The device shows minimal hysteresis and a near‐ideal subthreshold swing of 80 mV per decade. (e,f) Reproduced with permission.^72^ 2014, American Chemical Society. g) Schematic diagram of the FET device structure and the transfer curve based on SnS_2–x_Se_x_ crystals with the Se content of 0.8. Reproduced with permission.^74^ 2013, American Institute of Physics. h) Optical image of a representative device based on the heterostructure. Scale bar = 5 μm.i) Energy band alignment of WSe_2_ and SnSe_2_. (h,i) Reproduced with permission.^244^ 2016, American Institute of Physics.

FET, one of the most elementary components, consists of drain–source metal contacts, dielectric layer (gate electrode), and semiconducting channel. The drain–source current is controlled by the gate voltage on the dielectric layer. High carrier mobility, high switching ratio and low subthreshold swing means high performance FET, which depends on the metal contacts,^247^ channel materials (thickness,^248^, ^249^ doping,^192^, ^250^, ^251^, ^252^, ^253^, ^254^ heterostructures^200^, ^208^), dielectric materials (back‐gate,^86^, ^255^ top‐gate,^256^ liquid gate^257^), and so forth. 2D GIVMCs based FETs have demonstrated exciting performance. In the case of mechanically cleaved SnS_2_ nanomembrane (thickness of ≈15 nm) based back‐gated FETs reported by Peng and co‐workers,^70^ the transistor showed a high on/off ratio exceeding 10^6^, as well as an impressive carrier mobility of ≈1 cm^2^ V^−1^ s^−1^ (**Figure** **8**a). In addition, Koester and co‐workers^73^ reported mechanical exfoliated SnSe_2_ flake (thickness of 84 nm) based back‐gated FETs with high drive current of 160 μA μm^−1^ at *T* = 300 K, while the carrier mobility increases from 8.6 cm^2^ V^−1^ s^−1^ at 300 K to 28 cm^2^ V^−1^ s^−1^ at 77 K. The conductance obtained at *V*_ds_ = 50 meV revealed a little activation of only 5.5 meV, suggesting the Ohmic contacts at the source and drain contacts. These primary results showed great potential of 2D GIVMCs as the building block for future nanoelectronics.

The performance of FETs based on 2DLMCs can be influenced by the surface of the channel and contacted substrate resulting in a variety of charge traps or carrier scattering centers. High‐κ dielectric materials such as Al_2_O_3_, and HfO_2_, etc., have been proposed to be beneficial for high performance FETs due to dielectric screening of coulomb scattering on charged impurities.^258^ Tang and co‐workers^71^ have constructed high performance top‐gated FETs based on mechanical exfoliated monolayer SnS_2_ employing high‐κ dielectric Al_2_O_3_ (≈35 nm by atomic layer deposition) as the top gate (Figure 8b). The transistor showed a high carrier mobility of ≈50 cm^2^ V^−1^ s^−1^ (Figure 8c), much higher than that of back‐gated counterparts (≈1 cm^2^ V^−1^ s^−1^). While the subthreshold swing of ≈10 V per decade was still larger than general top‐gated FETs based on ultrathin nanoflakes. They speculated that the large subthreshold swing may be correlated with the nonefficient dielectric layer deposited at low temperature of 150 °C, which was limited by the lift‐off process or by shallow traps induced by iodine during transportation. The mobility had been improved by at least one order of magnitude compared with the back‐gated FETs based on few‐layer SnS_2_,^70^ suggesting the vital role of dielectric screening to the coulomb scattering.

None of the metal‐2DLMCs contacts are totally Ohmic resulting in the Schottky barriers in the interface, which causes low mobility due to the high contact resistance.^27^ Ionic liquid gate has been proposed to be an effective way to alleviate this problem. Gao and co‐workers.^121^ introduced a top capping layer of polymer electrolyte (anhydrous methanol solution of polyethylene oxide (PEO) containing 20 wt% LiClO_4_) combined with a back gate of 70 nm HfO_2_ (Figure 8d) to achieve high electron density over 10^13^ cm^−2^ based on few‐layer SnSe_2_, as well as a high on/off ratio of 10^4^, which was improve by two orders of magnitude compares with that of only back‐gated counterparts. Sutter and co‐workers^72^ obtained a high mobility of 230 cm^2^ V^−1^ s^−1^ based on few‐layer SnS_2_ FET employing deionized water as the top gate, as well as a minimal hysterisis of 15 mV as the top gate voltage swept in opposite directions (Figure 8e,f). This high performance may come from the absence of surface adsorbates in the solution environment and a valid screening of Coulumb scattering at the interface by the high‐κ dielectric. Peng and co‐workers^74^ have reported FETs based on mechanical exfoliated few‐layer SnS_2–x_Se_x_ with different Se content (Figure 8g). They found that the modulation by the gate voltage has been suppressed with the selenium content increasing.

In addition, there are many attempts to research heterostructures composed of 2DLMCs due to the novel physical properties. Xing and co‐workers^243^ for the first time demonstrated a room temperature Esaki tunnel diodes based on exfoliated p‐type black phosphorous (BP) and n‐type tin diselenide (SnSe_2_) which possess a broken‐gap energy band offset. Javey and co‐workers^244^ further fabricated 2D–2D tunneling FETs based on exfoliated WSe_2_ and SnSe_2_ heterostructures employing ZrO_2_ as the gate dielectric, allowing the scaling of gate oxide to improve the subthreshold swing of the device (Figure 8h,i). They presented high performance 2D–2D tunneling FETs such as the subthreshold swing of 100 meV and the maximum switching ratio *I*_on_/*I*_off_ of 10^7^. What is more, Rouvimov and co‐workers^246^ have reported the growth of multiple WS_2_/SnS layered heterostructures by atomic layer deposition, and the heterojunction based FET exhibited an ambipolar behavior with the electron mobility higher than that of WS_2_ based FETs.

### 4.2 Photodetectors Based on 2D GIVMCs

As illustrated in Table 1, the bandgaps of the GIVMCs varies in a wide range from ≈0.9 to ≈3.4 eV, and are calculated to have indirect and direct bandgaps in the bulk counterparts. Notably, SnS has been reported to have a high absorption coefficient (α > 10^4^ cm^−1^) across the direct absorption edge at 1.3–1.5 eV, rendering it as a promising candidate infrare photodetectors.^79^ What is more, GeSe and SnSe have been calculated to display extremely strong optical absorbance in the visible range as large as 47% when the thickness is down to monolayer or bilayer. Additionally, GeSe has a direct bandgap at monolayer or bilayer.^103^ There are many reports on the photodetectors based on 2D GIVMCs showing high performance as summarized in **Table** **2**.^75^, ^92^, ^93^, ^94^, ^95^, ^120^, ^130^, ^142^, ^149^ We will review some typical photodetectors in this section.

**Table 2:** Summary of typical 2D GIVMCs based photodetectors. (ML: multilayer; BL: bilayer; IR: infrared; WL: white light)

Device	Spectral range	*V*_ds_/*V*_g_[V]	Responvity[A W^−1^]	Rise time[ms]	Decay time[ms]	References
ML GeS	Visible	10/0	206	7	2.9 × 10^3^	92
ML GeSe	IR	5	3.5	100	3.6 × 10^3^	93
ML SnS_2_	Visible	–	100	44	44	72
ML SnS_2_	Visible	2/0	8.8 × 10^−3^	5 × 10^−3^	7 × 10^−3^	75
ML SnS_2_	Visible	10	2	42	42	149
ML SnS_2_	Visible	3/50	100	330	130	142
ML SnS_2_	Visible	1	1.5	42	40	186
ML SnS_2_	Visible	1	261	20	16	118
ML SnS_2_	Visible	5	3.4 × 10^−5^	400	200	147
ML SnSe	WL	0.1	330	–	–	105
ML SnSe_2_	IR	1/0	1.9	–	–	122
BL SnSe_2_	Visible	0.1	0.5	2.1	3.2	120
ML SnSe_2_	Visible	3	1.1 × 10^3^	14.5	8.1	76
ML SnSSe	Visible	10/0	4484	9	8	197

**Figure 9:**
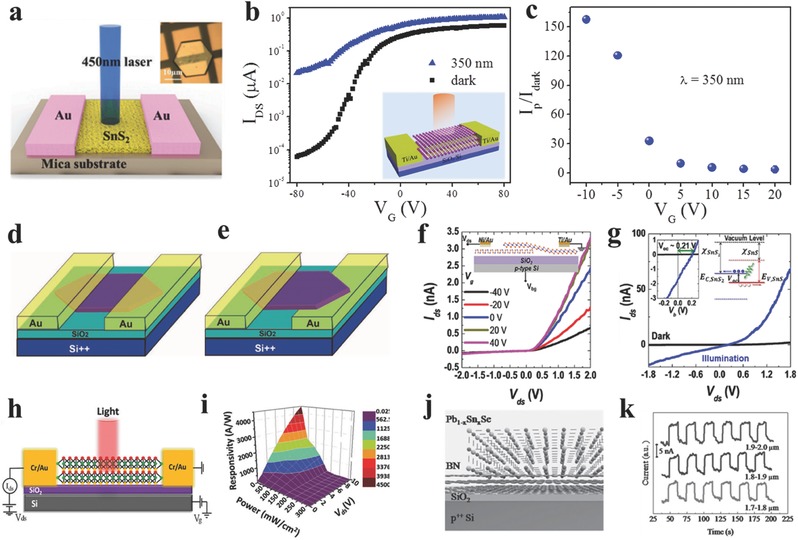
a) Schematic view of a SnS_2_ photodetector with 450 nm laser for illumination. Inset shows the optical image of a device. Reproduced with permission.^149^ b) Transfer curves at light illumination. Inset: schematic diagram of the phototransistor. c) Photoresponse ratio as a function of *V*_g_. (b,c) Reproduced with permission.^118^ d,e) Schematic diagrams of two kinds of single micrometer sized GeSe based photodetector with top‐contact and bottom‐up‐contact. Reproduced with permission.^94^ f) Gate tunable output characteristics of SnS_2_/SnS vertical heterostructures, inset: device schematic. g) Dark *I–V* curve and *I*_ph_–*V* curve under 3.06 eV light illumination with a power of 3.2 μW. Left inset: photovoltaic *I*–*V* curves, showing the open‐circuit voltage of 0.21 V. Right inset: corresponding band diagram of the heterojunctions. f,g) Reproduced with permission.^130^ 2015, American Chemical Society. h,i) Schematic of the SnS_2–x_Se_x_ based photodetectors, and 3D view of photoresponsivity mapping of few‐layered SnS_2–x_Se_x_ phototransistor. Reproduced with permission.^197^ 2016, American Chemical Society. j,k) Schematic diagram for epitaxial growth of Pb_1–x_Sn_x_Se nanoplates on few‐layer BN, and mid‐infrared detection of Pb_1–x_Sn_x_Se nanoplates at 1.7–2.0 μm. Reproduced with permission.^107^

The typical photodetector based on 2D SnS_2_ crystal has been demonstrated by Meng and co‐workers^149^ showing a fast response time of 42 ms while the low responsivity of 2 A W^−1^ (**Figure** **9**a). Zhai and co‐workers^76^ fabricated a high performance photodetector based on few‐layer SnSe_2_ flake with a high responsivity of 1.1 × 10^3^ A W^−1^, as well as a fast response time of 8.1 ms due to the high‐quality and ultrathin thickness. Furthermore, Zhai and co‐workers^118^ constructed high performance phototransistors based on ultrathin SnS_2_ nanosheets with a high responsivity of 261 A W^−1^ and fast response time of 16 ms, they systematically studied the modulation of back‐gate voltage on the photodetection behaviors. As shown in Figure 9b, photocurrents increased for both On and Off states at the whole process, suggesting that the photocurrents govern the whole process over thermionic and tunneling currents. And they obtained a high sensitivity of 150 at *V*_g_ = −10 V (Figure 9c), while a high responsivity of 400 A W^−1^ achieved at *V*_g_ = 20 V, showing the promising applications in the pixelated imaging systems by such excellent gate‐tunable photoresponse. Wan and co‐workers^94^ studied the anisotropic photoresponse of GeSe nanosheets based photodetectors (Figure 9d,e). The carrier transport in direction parallel to the layers is in the intralayer through covalent bonds, while the vertical transport happens via hopping. According to the calculations, the effective mass of holes in the direction vertical to the layers is larger than that in the parallel direction, resulting in the anisotropic dark current.

Jo and co‐workers^130^ synthesized polymorphic 2D tin‐sulfides of either p‐type SnS or n‐type SnS_2_ via adjusting hydrogen during the process. Then they fabricated p‐n heterostructures based on synthesized SnS and SnS_2_ and showed typical rectifying characteristic (Figure 9f). The responsivity has been improved from 4.56 mA W^−1^ at forward bias to 27.09 mA W^−1^ at reverse bias (Figure 9g). Chen and co‐workers^197^ fabricated a high performance phototransistor based on mechanical exfoliated few‐layer SnS_2–x_Se_x_ with a high photoresponsivity of 6000 A W^−1^ at *V*_g_ = 80 V and a fast response time of 9 ms (Figure 9h,i). They excluded the photoconductivity dominating the process, which results in a very slow response time not consistent with their fast response results. Then they speculated that the high‐quality single line structure with low defect density is crucial role in the process, confirmed by the small hysterisis and the pronounced XRD spectrum. Recently, He and co‐workers^107^ directly grown ultrathin Pb_1–x_Sn_x_Se nanoplates on BN of which the surface is free of dangling bonds allowing the direct growth of a highly lattice‐mismatched heterosturctures (Figure 9j). Therefore, the relaxed strain and less surface states at the interface of epilayer and BN can not only improve their electronic properties but also promote their applications in integration techniques. Because of its narrow direct bandgap, the photodetectors based on ultrathin Pb_1–x_Sn_x_Se nanoplates grown on BN demonstrated high‐efficient response to mid‐infrared light (1.7–2.0 μm) (Figure 9k), indicating their promising applications in environmental monitoring, remote sensing, military communication, and so on.

### 4.3 Flexible Photodetectors Based on 2D GIVMCs

One of the most advantages of 2DLMCs is the flexibility and compatibility with flexible devices. There are also many reports on the flexible electronics based on 2D GIVMCs.^104^, ^105^, ^118^, ^197^, ^259^ In the following context, we will present some typical flexible devices based on 2D GIVMCs.

**Figure 10:**
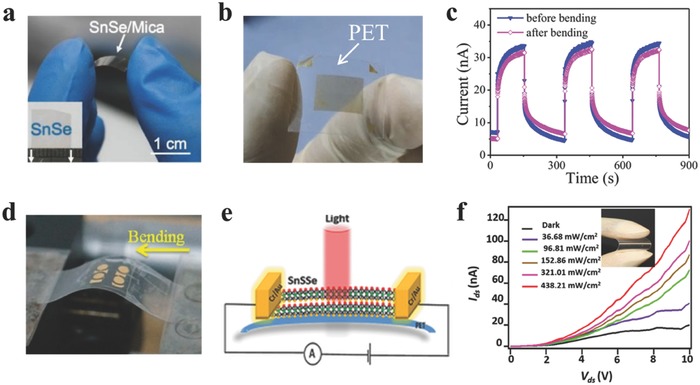
a) Optical image of the SnSe nanoplates based photodetectors on flexible mica sheets. Reproduced with permission.^105^ 2015, Springer. b,c) Optical image of the SnS_2_ nanosheets based devices on flexible PET film, and the *I–t* curves before and after bending the PET film. Reproduced with permission.^118^ d) Photograph of instrument used for bending. Reproduced with permission.^104^ 2015, American Chemical Society. e,f) Schematic diagram of SnS_2–x_Se_x_ phototransistor on PET, and photoconductivity measurements of flexible SnS_2–x_Se_x_ phototransistor on PET. Reproduced with permission.^197^

Liu and co‐workers^105^ fabricated SnSe nanoplates based photodetectors on flexible mica through PVD method (**Figure** **10**a), with a high responsivity of 330 A W^−1^ under white light illumination. Zhai and co‐workers^118^ have successfully transferred the as‐synthesized ultrathin SnS_2_ nanosheets onto flexible polyethylene terephthalate (PET) film (Figure 10b). The constructed photodetector on PET films showed high responsivity of 34.6 A W^−1^, and still remained 26.9 A W^−1^ after bending 200 times (Figure 10c), showing good flexibility. What is more, the 2D ternary alloys showing excellent performance for electroncis and optoelectroncis^74^, ^107^ have also been applied to flexible photodetectors. For example, He and co‐workers^104^ have reported ultrathin Pb_1–x_Sn_x_Se nanoplates based photodetectors on mica sheets showing broad spectra detection from UV to infrared light (Figure 10d). Additionally, Chen and co‐workers^197^ constructed few‐layer SnS_2–x_Se_x_ with excellent photoresponse under varied light illumination power density (Figure 10e,f).

## 5 Conclusions and Outlook

**Figure 11:**
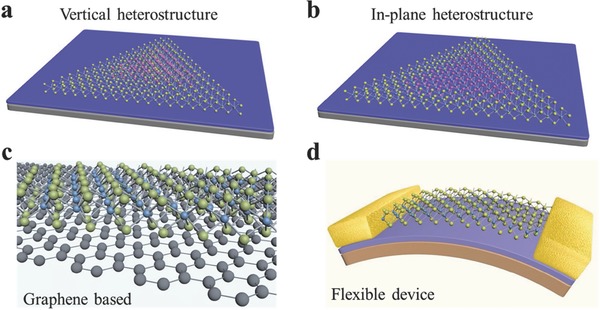
a,b) Schematic diagrams of the in situ growth vertical and in‐plane heterostructure. c) Illustration of the hybrid structures based on graphene and 2D GIVMCs. d) Schematic of the flexible devices based on 2D GIVMCs.

In this review, we have presented typical fundamental properties and the recent advancements on the synthesis of 2D GIVMCs and their applications in electronics and optoelectronics. These reports on 2D GIVMCs have shown promising applications in next‐generation electric, optical, and photonic systems, the research on 2D GIVMCs is still in the preliminary stage which is mainly restricted on the mechanical exfoliated nanosheets. Although mechanical cleavage method is fairly simple and easy to obtain high quality nanoflakes, the extremely low yield, and the low controllability of the layer number and large‐area uniformity have harshly restricted it from practical applications in electronics and optoelectronics. In contrast, CVD is a promising route for high‐quality and large‐area 2D GIVMCs flakes which is desirable for current electronic industry. However, the CVD growth of atomically 2D GIVMCs is in the initiate stage and remains great challenge in the future. Therefore, there is still a huge room for further study on the controlled synthesis of high‐quality and large‐scale single crystalline 2D GIVMCs. Moreover, in‐situ growth of vertical (**Figure** **11**a) and lateral (Figure 11b) heterostructures are very important for exploring the novel physical properties, considering that most of the reports on 2D GIVMCs is currently based on the transferred heterojunctions,^130^, ^243^, ^244^ which may harm the electronic properties^231^, ^240^ due to the dangling bonds and adsorbates at the interface. In addition, graphene is an ideal template to promote the nucleation and growth of other 2DLMCs crystals for producing functional hybrid structures via CVD method (Figure 11c),^198^, ^204^, ^207^ which may result in the modulation of the electric and optical properties coupled with graphene. The fast growing field calls for more works to fully understand the junction formation and resulted physical properties in 2D GIVMCs crystals. Besides the efforts on the study of electronics and optoelectronics, extension of the applications of 2D GIVMCs based devices is another important issue. For example, the flexible electronics and optoelectronics (Figure 11d) can be further explored for the applications in remote sensing, fiber‐optic communications, military communications, biological imaging, and so on. More importantly, all Ge‐chalcogenides (S, Se) are p‐type semiconductors, which are desirable for fully exploring the electronics. However, there are few reports on these materials especially for ultrathin structures. In a word, the recent exciting achievements on the study of 2D GIVMCs have demonstrated their great advantages in next‐generation electronics and optoelectronics.

## References

[1] K. S. Novoselov , A. K. Geim , S. V. Morozov , D. Jiang , Y. Zhang , S. V. Dubonos , I. V. Grigorieva , A. A. Firsov , Science 2004, 306, 666. 15499015 10.1126/science.1102896

[2] G. R. Bhimanapati , Z. Lin , V. Meunier , Y. Jung , J. Cha , S. Das , D. Xiao , Y. Son , M. S. Strano , V. R. Cooper , L. Liang , S. G. Louie , E. Ringe , W. Zhou , S. S. Kim , R. R. Naik , B. G. Sumpter , H. Terrones , F. Xia , Y. Wang , J. Zhu , D. Akinwande , N. Alem , J. A. Schuller , R. E. Schaak , M. Terrones , J. A. Robinson , ACS Nano 2015, 9, 11509. 26544756 10.1021/acsnano.5b05556

[3] J. Choi , H. Zhang , J. H. Choi , ACS Nano 2016, 10, 1671. 26720839 10.1021/acsnano.5b07457

[4] X. Ling , Y. Lin , Q. Ma , Z. Wang , Y. Song , L. Yu , S. Huang , W. Fang , X. Zhang , A. L. Hsu , Y. Bie , Y. H. Lee , Y. Zhu , L. Wu , J. Li , P. Jarillo‐Herrero , M. Dresselhaus , T. Palacios , J. Kong , Adv. Mater. 2016, 28, 2322. 26813882 10.1002/adma.201505070

[5] M. Buscema , J. O. Island , D. J. Groenendijk , S. I. Blanter , G. A. Steele , H. S. van der Zant , A. Castellanos‐Gomez , Chem. Soc. Rev. 2015, 44, 3691. 25909688 10.1039/c5cs00106d

[6] X. Duan , C. Wang , A. Pan , R. Yu , Chem. Soc. Rev. 2015, 44, 8859. 26479493 10.1039/c5cs00507h

[7] X. Huang , Z. Zeng , H. Zhang , Chem. Soc. Rev. 2013, 42, 1934. 23344899 10.1039/c2cs35387c

[8] Q. Ji , Y. Zhang , Z. Liu , Chem. Soc. Rev. 2015, 44, 2587. 25256261 10.1039/c4cs00258j

[9] G. B. Liu , D. Xiao , Y. Yao , X. Xu , W. Yao , Chem. Soc. Rev. 2015, 44, 2643. 25474725 10.1039/c4cs00301b

[10] Y. Shi , H. Li , L. J. Li , Chem. Soc. Rev. 2015, 44, 2744. 25327436 10.1039/c4cs00256c

[11] H. Wang , H. Yuan , S. Sae Hong , Y. Li , Y. Cui , Chem. Soc. Rev. 2015, 44, 2664. 25474482 10.1039/c4cs00287c

[12] W. Huang , L. Gan , H. Li , Y. Ma , T. Zhai , CrystEngComm 2016, 18, 3968.

[13] B. Sa , Z. Sun , B. Wu , Nanoscale 2015, 8, 1169. 10.1039/c5nr06871a 26667941

[14] H. Wang , F. Liu , W. Fu , Z. Fang , W. Zhou , Z. Liu , Nanoscale 2014, 6, 12250. 25219598 10.1039/c4nr03435j

[15] M. Chhowalla , H. S. Shin , G. Eda , L. J. Li , K. P. Loh , H. Zhang , Nat. Chem. 2013, 5, 263. 23511414 10.1038/nchem.1589

[16] M. Liu , X. Yin , E. Ulin‐Avila , B. Geng , T. Zentgraf , L. Ju , F. Wang , X. Zhang , Nature 2011, 474, 64. 21552277 10.1038/nature10067

[17] L. Gao , G. X. Ni , Y. Liu , B. Liu , A. H. Castro Neto , K. P. Loh , Nature 2014, 505, 190. 24336218 10.1038/nature12763

[18] W. Zhang , J. K. Huang , C. H. Chen , Y. H. Chang , Y. J. Cheng , L. J. Li , Adv. Mater. 2013, 25, 3456. 23703933 10.1002/adma.201301244

[19] G. Dubey , R. Urcuyo , S. Abb , G. Rinke , M. Burghard , S. Rauschenbach , K. Kern , J. Am. Chem. Soc. 2014, 136, 13482. 25185758 10.1021/ja5046499

[20] B. Chitara , L. S. Panchakarla , S. B. Krupanidhi , C. N. Rao ,Adv. Mater. 2011, 23, 5419. 21786342 10.1002/adma.201101414

[21] J. Yin , H. Wang , H. Peng , Z. Tan , L. Liao , L. Lin , X. Sun , A. L. Koh , Y. Chen , H. Peng , Z. Liu , Nat. Commun. 2016, 7, 10699. 26948537 10.1038/ncomms10699 PMC4786639

[22] X. Wang , J. B. Xu , C. Wang , J. Du , W. Xie , Adv. Mater. 2011, 23, 2464. 21484896 10.1002/adma.201100476

[23] D. Sinha , J. U. Lee , Nano Lett. 2014, 14, 4660. 25000510 10.1021/nl501735k

[24] M. T. Mihnev , J. R. Tolsma , C. J. Divin , D. Sun , R. Asgari , M. Polini , C. Berger , W. A. de Heer , A. H. MacDonald , T. B. Norris ,Nat. Commun. 2015, 6, 8105. 26399955 10.1038/ncomms9105 PMC4598362

[25] K. F. Mak , C. Lee , J. Hone , J. Shan , T. F. Heinz , Phys. Rev. Lett. 2010, 105, 136805. 21230799 10.1103/PhysRevLett.105.136805

[26] D. Lembke , S. Bertolazzi , A. Kis , Acc. Chem. Res. 2015, 48, 100. 25555202 10.1021/ar500274q

[27] Y. P. Venkata Subbaiah , K. J. Saji , A. Tiwari , Adv. Funct. Mater. 2016, 26, 2046.

[28] T. Cheiwchanchamnangij , W. R. L. Lambrecht , Phys. Rev. B 2012, 85, 115317.

[29] Z. He , W. Xu , Y. Zhou , X. Wang , Y. Sheng , Y. Rong , S. Guo , J. Zhang , J. M. Smith , J. H. Warner , ACS Nano 2016, 10, 2176. 26761127 10.1021/acsnano.5b06678

[30] A. L. Elias , N. Perea‐Lopez , A. Castro‐Beltran , A. Berkdemir , R. Lv , S. Feng , A. D. Long , T. Hayashi , Y. A. Kim , M. Endo , H. R. Gutierrez , N. R. Pradhan , L. Balicas , T. E. Mallouk , F. Lopez‐Urias , H. Terrones , M. Terrones , ACS Nano 2013, 7, 5235. 23647141 10.1021/nn400971k

[31] S. J. Yun , S. H. Chae , H. Kim , J. C. Park , J. H. Park , G. H. Han , J. S. Lee , S. M. Kim , H. M. Oh , J. Seok , M. S. Jeong , K. K. Kim , Y. H. Lee , ACS Nano 2015, 9, 5510. 25873415 10.1021/acsnano.5b01529

[32] Y. Zhang , Q. Ji , J. Ju , H. Yuan , J. Shi , T. Gao , D. Ma , M. Liu , Y. Chen , X. Song , H. Y. Hwang , Y. Cui , Z. Liu , ACS Nano 2013, 7, 8963. 24047054 10.1021/nn403454e

[33] N. Perea‐López , A. L. Elías , A. Berkdemir , A. Castro‐Beltran , H. R. Gutiérrez , S. Feng , R. Lv , T. Hayashi , F. López‐Urías , S. Ghosh , B. Muchharla , S. Talapatra , H. Terrones , M. Terrones , Adv. Funct. Mater. 2013, 23, 5511.

[34] H. R. Gutierrez , N. Perea‐Lopez , A. L. Elias , A. Berkdemir , B. Wang , R. Lv , F. Lopez‐Urias , V. H. Crespi , H. Terrones , M. Terrones ,Nano Lett. 2013, 13, 3447. 23194096 10.1021/nl3026357

[35] A. Chernikov , C. Ruppert , H. M. Hill , A. F. Rigosi , T. F. Heinz ,Nat. Photonics 2015, 9, 466.

[36] S. Zhou , J. Chen , L. Gan , Q. Zhang , Z. Zheng , H. Li , T. Zhai ,Sci. Bull. 2016, 61, 227.

[37] J. Yuan , J. Wu , W. J. Hardy , P. Loya , M. Lou , Y. Yang , S. Najmaei , M. Jiang , F. Qin , K. Keyshar , H. Ji , W. Gao , J. Bao , J. Kono , D. Natelson , P. M. Ajayan , J. Lou , Adv. Mater. 2015, 27, 5605. 26293810 10.1002/adma.201502075

[38] W. Feng , W. Zheng , W. Cao , P. Hu , Adv. Mater. 2014, 26, 6587. 25167845 10.1002/adma.201402427

[39] G. W. Mudd , S. A. Svatek , T. Ren , A. Patane , O. Makarovsky , L. Eaves , P. H. Beton , Z. D. Kovalyuk , G. V. Lashkarev , Z. R. Kudrynskyi , A. I. Dmitriev , Adv. Mater. 2013, 25, 5714. 23966225 10.1002/adma.201302616 PMC4065344

[40] J. O. Island , S. I. Blanter , M. Buscema , H. S. J. van der Zant , A. Castellanos‐Gomez , Nano Lett. 2015, 15, 7853. 26540135 10.1021/acs.nanolett.5b02523

[41] D. Wu , A. J. Pak , Y. Liu , Y. Zhou , X. Wu , Y. Zhu , M. Lin , Y. Han , Y. Ren , H. Peng , Y.‐H. Tsai , G. S. Hwang , K. Lai , Nano Lett. 2015, 15, 8136. 26575786 10.1021/acs.nanolett.5b03575

[42] J. Zhou , Q. Zeng , D. Lv , L. Sun , L. Niu , W. Fu , F. Liu , Z. Shen , C. Jin , Z. Liu , Nano Lett. 2015, 15, 6400. 26360543 10.1021/acs.nanolett.5b01590

[43] P. Hu , Z. Wen , L. Wang , P. Tan , K. Xiao , ACS Nano 2012, 6, 5988. 22676041 10.1021/nn300889c

[44] X. Li , L. Basile , B. Huang , C. Ma , J. Lee , I. V. Vlassiouk , A. A. Puretzky , M. W. Lin , M. Yoon , M. Chi , J. C. Idrobo , C. M. Rouleau , B. G. Sumpter , D. B. Geohegan , K. Xiao , ACS Nano 2015, 9, 8078. 26202730 10.1021/acsnano.5b01943

[45] D. J. Late , B. Liu , J. Luo , A. Yan , H. S. Matte , M. Grayson , C. N. Rao , V. P. Dravid , Adv. Mater. 2012, 24, 3549. 22678832 10.1002/adma.201201361

[46] W. Kim , C. Li , F. A. Chaves , D. Jiménez , R. D. Rodriguez , J. Susoma , M. A. Fenner , H. Lipsanen , J. Riikonen , Adv. Mater. 2016, 28, 1845. 26727653 10.1002/adma.201504514

[47] X. Li , L. Basile , M. Yoon , C. Ma , A. A. Puretzky , J. Lee , J. C. Idrobo , M. Chi , C. M. Rouleau , D. B. Geohegan , K. Xiao , Angew. Chem. Int. Ed. 2015, 54, 2712. 10.1002/anie.201409743 25611050

[48] S. Lei , L. Ge , Z. Liu , S. Najmaei , G. Shi , G. You , J. Lou , R. Vajtai , P. M. Ajayan , Nano Lett. 2013, 13, 2777. 23731066 10.1021/nl4010089

[49] M. Lin , D. Wu , Y. Zhou , W. Huang , W. Jiang , W. Zheng , S. Zhao , C. Jin , Y. Guo , H. Peng , Z. Liu , J. Am. Chem. Soc. 2013, 135, 13274. 23978251 10.1021/ja406351u

[50] S. Lei , F. Wen , L. Ge , S. Najmaei , A. George , Y. Gong , W. Gao , Z. Jin , B. Li , J. Lou , J. Kono , R. Vajtai , P. Ajayan , N. J. Halas ,Nano Lett. 2015, 15, 3048. 25822539 10.1021/acs.nanolett.5b00016

[51] W. Zheng , T. Xie , Y. Zhou , Y. L. Chen , W. Jiang , S. Zhao , J. Wu , Y. Jing , Y. Wu , G. Chen , Y. Guo , J. Yin , S. Huang , H. Q. Xu , Z. Liu , H. Peng , Nat. Commun. 2015, 6, 6972. 25898022 10.1038/ncomms7972 PMC4411293

[52] F. Liu , H. Shimotani , H. Shang , T. Kanagasekaran , V. Zolyomi , N. Drummond , V. I. Fal'ko , K. Tanigaki , ACS Nano 2014, 8, 752. 24364508 10.1021/nn4054039

[53] Z. Wang , K. Xu , Y. Li , X. Zhan , M. Safdar , Q. Wang , F. Wang , J. He , ACS Nano 2014, 8, 4859. 24697541 10.1021/nn500782n

[54] E. Liu , M. Long , J. Zeng , W. Luo , Y. Wang , Y. Pan , W. Zhou , B. Wang , W. Hu , Z. Ni , Y. You , X. Zhang , S. Qin , Y. Shi , K. Watanabe , T. Taniguchi , H. Yuan , H. Y. Hwang , Y. Cui , F. Miao , D. Xing , Adv. Funct. Mater. 2016, 26, 1938.

[55] F. Liu , S. Zheng , X. He , A. Chaturvedi , J. He , W. L. Chow , T. R. Mion , X. Wang , J. Zhou , Q. Fu , H. J. Fan , B. K. Tay , L. Song , R.‐H. He , C. Kloc , P. M. Ajayan , Z. Liu , Adv. Funct. Mater. 2016, 26, 1169.

[56] E. Zhang , Y. Jin , X. Yuan , W. Wang , C. Zhang , L. Tang , S. Liu , P. Zhou , W. Hu , F. Xiu , Adv. Funct. Mater. 2015, 25,4076.

[57] K. Xu , Z. Wang , F. Wang , Y. Huang , F. Wang , L. Yin , C. Jiang , J. He , Adv. Mater. 2015, 27, 7881. 26497945 10.1002/adma.201503864

[58] O. Lopez‐Sanchez , D. Lembke , M. Kayci , A. Radenovic , A. Kis , Nat. Nanotechnol. 2013, 8, 497. 23748194 10.1038/nnano.2013.100

[59] S. Cui , H. Pu , S. A. Wells , Z. Wen , S. Mao , J. Chang , M. C. Hersam , J. Chen , Nat. Commun. 2015, 6, 8632. 26486604 10.1038/ncomms9632 PMC4639804

[60] C. C. Mayorga‐Martinez , Z. Sofer , M. Pumera , Angew. Chem. Int. Ed. 2015, 54, 14317. 10.1002/anie.201505015 26403872

[61] E. Liu , Y. Fu , Y. Wang , Y. Feng , H. Liu , X. Wan , W. Zhou , B. Wang , L. Shao , C. H. Ho , Y. S. Huang , Z. Cao , L. Wang , A. Li , J. Zeng , F. Song , X. Wang , Y. Shi , H. Yuan , H. Y. Hwang , Y. Cui , F. Miao , D. Xing , Nat. Commun. 2015, 6, 6991. 25947630 10.1038/ncomms7991 PMC4432591

[62] J. Miao , S. Zhang , L. Cai , M. Scherr , C. Wang , ACS Nano 2015, 9, 9236. 26277886 10.1021/acsnano.5b04036

[63] H. Yuan , G. Cheng , L. You , H. Li , H. Zhu , W. Li , J. J. Kopanski , Y. S. Obeng , A. R. Hight Walker , D. J. Gundlach , C. A. Richter , D. E. Ioannou , Q. Li , ACS Appl. Mater. Interfaces 2015, 7, 1180. 25514512 10.1021/am506921y

[64] J. Y. Kwak , J. Hwang , B. Calderon , H. Alsalman , N. Munoz , B. Schutter , M. G. Spencer , Nano Lett. 2014, 14, 4511. 24978093 10.1021/nl5015316

[65] C. Huo , Z. Yan , X. Song , H. Zeng , Sci. Bull. 2015, 60, 1994.

[66] S. R. Tamalampudi , Y. Y. Lu , U. R. Kumar , R. Sankar , C. D. Liao , B. K. Moorthy , C. H. Cheng , F. C. Chou , Y. T. Chen , Nano Lett. 2014, 14, 2800. 24742243 10.1021/nl500817g

[67] P. Hu , L. Wang , M. Yoon , J. Zhang , W. Feng , X. Wang , Z. Wen , J. C. Idrobo , Y. Miyamoto , D. B. Geohegan , K. Xiao , Nano Lett. 2013, 13, 1649. 23465066 10.1021/nl400107k

[68] Z. Wang , M. Safdar , M. Mirza , K. Xu , Q. Wang , Y. Huang , F. Wang , X. Zhan , J. He , Nanoscale 2015, 7, 7252. 25811647 10.1039/c4nr07313d

[69] X. Zhou , L. Gan , Q. Zhang , X. Xiong , H. Li , Z. Zhong , J. Han , T. Zhai , J. Mater. Chem. C 2016, 4, 2111.

[70] D. De , J. Manongdo , S. See , V. Zhang , A. Guloy , H. Peng , Nanotechnology 2013, 24, 025202. 23238583 10.1088/0957-4484/24/2/025202

[71] H. S. Song , S. L. Li , L. Gao , Y. Xu , K. Ueno , J. Tang , Y. B. Cheng , K. Tsukagoshi , Nanoscale 2013, 5, 9666. 23989804 10.1039/c3nr01899g

[72] Y. Huang , E. Sutter , J. T. Sadowski , M. Cotlet , O. L. Monti , D. A. Racke , M. R. Neupane , D. Wickramaratne , R. K. Lake , B. A. Parkinson , P. Sutter , ACS Nano 2014, 8, 10743. 25247490 10.1021/nn504481r

[73] Y. Su , M. A. Ebrish , E. J. Olson , S. J. Koester , Appl. Phys. Lett. 2013, 103, 263104.

[74] T. S. Pan , D. De , J. Manongdo , A. M. Guloy , V. G. Hadjiev , Y. Lin , H. B. Peng , Appl. Phys. Lett. 2013, 103, 093108.

[75] G. Su , V. G. Hadjiev , P. E. Loya , J. Zhang , S. Lei , S. Maharjan , P. Dong , M. A. P , J. Lou , H. Peng , Nano Lett. 2015, 15, 506. 25494406 10.1021/nl503857r

[76] X. Zhou , L. Gan , W. Tian , Q. Zhang , S. Jin , H. Li , Y. Bando , D. Golberg , T. Zhai , Adv. Mater. 2015, 27, 8035. 26541236 10.1002/adma.201503873

[77] C. Kamal , A. Chakrabarti , M. Ezawa , Phys. Rev. B 2016, 93, 125428.

[78] P. Sinsermsuksakul , R. Chakraborty , S. B. Kim , S. M. Heald , T. Buonassisi , R. G. Gordon , Chem. Mater. 2012, 24, 4556.

[79] J. Xia , X. Z. Li , X. Huang , N. Mao , D. D. Zhu , L. Wang , H. Xu , X. M. Meng , Nanoscale 2016, 8, 2063. 26698370 10.1039/c5nr07675g

[80] M. Micoulaut , W. Wełnic , M. Wuttig , Phys. Rev. B 2008, 78, 224209.

[81] R. Y. Wang , M. A. Caldwell , R. G. D. Jeyasingh , S. Aloni , R. M. Shelby , H. S. P. Wong , D. J. Milliron , J. Appl. Phys. 2011, 109, 113506.

[82] K.‐M. Chung , D. Wamwangi , M. Woda , M. Wuttig , W. Bensch ,J. Appl. Phys. 2008, 103, 083523.

[83] Y. Huang , C. Ling , H. Liu , S. Wang , B. Geng , J. Phys. Chem. C 2014, 118, 9251.

[84] Z. Yin , H. Li , L. Jiang , Y. Shi , Y. Sun , G. Lu , Q. Zhang , X. Chen , H. Zhang , ACS Nano 2012, 6, 74. 22165908 10.1021/nn2024557

[85] X. Wang , Y. Gong , G. Shi , W. L. Chow , K. Keyshar , G. Ye , R. Vajtai , J. Lou , Z. Liu , E. Ringe , B. K. Tay , P. M. Ajayan , ACS Nano 2014, 8, 5125. 24680389 10.1021/nn501175k

[86] W. Feng , W. Zheng , W. Cao , P. Hu , Adv. Mater. 2014, 26, 6587. 25167845 10.1002/adma.201402427

[87] S. Fathipour , N. Ma , W. S. Hwang , V. Protasenko , S. Vishwanath , H. G. Xing , H. Xu , D. Jena , J. Appenzeller , A. Seabaugh , Appl. Phys. Lett. 2014, 105, 192101.

[88] L. Zhou , K. Xu , A. Zubair , A. D. Liao , W. Fang , F. Ouyang , Y. H. Lee , K. Ueno , R. Saito , T. Palacios , J. Kong , M. S. Dresselhaus , J. Am. Chem. Soc. 2015, 137, 11892. 26305492 10.1021/jacs.5b07452

[89] Z. Shi , Z. Zhang , A. Kutana , B. I. Yakobson , ACS Nano 2015, 9, 9802. 26394207 10.1021/acsnano.5b02753

[90] J. H. Yang , Y. Zhang , W. J. Yin , X. G. Gong , B. I. Yakobson , S. H. Wei , Nano Lett. 2016, 16, 1110. 26741149 10.1021/acs.nanolett.5b04341

[91] L. Shi , Y. M. Dai , J. Appl. Crystallogr. 2014, 47, 527.

[92] R. K. Ulaganathan , Y. Y. Lu , C. J. Kuo , S. R. Tamalampudi , R. Sankar , K. M. Boopathi , A. Anand , K. Yadav , R. J. Mathew , C. R. Liu , F. C. Chou , Y. T. Chen , Nanoscale 2016, 8, 2284. 26743029 10.1039/c5nr05988g

[93] B. Mukherjee , Y. Cai , H. R. Tan , Y. P. Feng , E. S. Tok , C. H. Sow , ACS Appl. Mater. Interfaces 2013, 5, 9594. 24028388 10.1021/am402550s

[94] D. J. Xue , J. Tan , J. S. Hu , W. Hu , Y. G. Guo , L. J. Wan , Adv. Mater. 2012, 24, 4528. 22806941 10.1002/adma.201201855

[95] S. M. Yoon , H. J. Song , H. C. Choi , Adv. Mater. 2010, 22, 2164. 20564254 10.1002/adma.200903719

[96] D. D. Vaughn , 2nd , R. J. Patel , M. A. Hickner , R. E. Schaak , J. Am. Chem. Soc. 2010, 132, 15170. 20942394 10.1021/ja107520b

[97] Z. Deng , D. Cao , J. He , S. Lin , S. M. Lindsay , Y. Liu , ACS Nano 2012, 6, 6197. 22738287 10.1021/nn302504p

[98] A. Rabkin , S. Samuha , R. E. Abutbul , V. Ezersky , L. Meshi , Y. Golan , Nano Lett. 2015, 15, 2174. 25710674 10.1021/acs.nanolett.5b00209

[99] Z. Mutlu , R. J. Wu , D. Wickramaratne , S. Shahrezaei , C. Liu , S. Temiz , A. Patalano , M. Ozkan , R. K. Lake , K. A. Mkhoyan , C. S. Ozkan , Small 2016, 12, 2998. 27099950 10.1002/smll.201600559

[100] M. B. Alemayehu , M. Falmbigl , K. Ta , D. C. Johnson , ACS Nano 2015, 9, 4427. 25853288 10.1021/acsnano.5b01770

[101] Z. Wang , J. Wang , Y. Zang , Q. Zhang , J. A. Shi , T. Jiang , Y. Gong , C. L. Song , S. H. Ji , L. L. Wang , L. Gu , K. He , W. Duan , X. Ma , X. Chen , Q. K. Xue , Adv. Mater. 2015, 27, 4150. 26058925 10.1002/adma.201501676

[102] L. Li , Z. Chen , Y. Hu , X. Wang , T. Zhang , W. Chen , Q. Wang , J. Am. Chem. Soc. 2013, 135, 1213. 23311291 10.1021/ja3108017

[103] G. Shi , E. Kioupakis , Nano Lett. 2015, 15, 6926. 26393677 10.1021/acs.nanolett.5b02861

[104] Q. Wang , K. Xu , Z. Wang , F. Wang , Y. Huang , M. Safdar , X. Zhan , Z. Cheng , J. He , Nano Lett. 2015, 15, 1183. 25603278 10.1021/nl504258m

[105] S. Zhao , H. Wang , Y. Zhou , L. Liao , Y. Jiang , X. Yang , G. Chen , M. Lin , Y. Wang , H. Peng , Z. Liu , Nano Res. 2015, 8, 288.

[106] J. Z. Jian Zhang , H. Zhu , X. Wu , H. Cui , D. Li , J. Jiang , C. Gao , Q. Wang , Q. Cui , Nanoscale 2015, 7, 10807. 26269801 10.1039/c5nr02131f

[107] Q. Wang , Y. Wen , F. Yao , Y. Huang , Z. Wang , M. Li , X. Zhan , K. Xu , F. Wang , J. Li , K. Liu , C. Jiang , F. Liu , J. He , Small 2015, 11, 5388. 26305343 10.1002/smll.201502049

[108] G. Han , S. R. Popuri , H. F. Greer , J. G. Bos , W. Zhou , A. R. Knox , A. Montecucco , J. Siviter , E. A. Man , M. Macauley , D. J. Paul , W. G. Li , M. C. Paul , M. Gao , T. Sweet , R. Freer , F. Azough , H. Baig , N. Sellami , T. K. Mallick , D. H. Gregory , Angew. Chem. Int. Ed. 2016, 55, 6433. 10.1002/anie.201601420 PMC5074331 27094703

[109] J. Shen , Y. Xie , J. J. Cha , Nano Lett. 2015, 15, 3827. 25938713 10.1021/acs.nanolett.5b00576

[110] Q. Wang , K. Cai , J. Li , Y. Huang , Z. Wang , K. Xu , F. Wang , X. Zhan , K. Wang , J. He , Adv. Mater. 2016, 28, 617. 26618500 10.1002/adma.201504630

[111] Q. Wang , S.‐Z. Kang , X. Li , Y.‐W. Yang , L. Qin , J. Mu , J. Alloys Compd. 2015, 631, 21.

[112] X. Wang , B. Liu , Q. Wang , W. Song , X. Hou , D. Chen , Y. B. Cheng , G. Shen , Adv. Mater. 2013, 25, 1479. 23180449 10.1002/adma.201204063

[113] B. Mukherjee , E. S. Tok , C. H. Sow , J. Appl. Phys. 2013, 114, 134302.

[114] B. Mukherjee , Z. Hu , M. Zheng , Y. Cai , Y. P. Feng , E. S. Tok , C. H. Sow , J. Mater. Chem. 2012, 22, 24882.

[115] L. Properzi , A. Di Cicco , L. Nataf , F. Baudelet , T. Irifune , Sci. Rep. 2015, 5, 10188. 25973778 10.1038/srep10188 PMC4650749

[116] H. Zang , P. K. Routh , Y. Huang , J. S. Chen , E. Sutter , P. Sutter , M. Cotlet , ACS Nano 2016, 10, 4790. 27031885 10.1021/acsnano.6b01538

[117] T. Zhou , W. K. Pang , C. Zhang , J. Yang , Z. Chen , H. K. Liu , Z. Guo , ACS Nano 2014, 8, 8323. 25010575 10.1021/nn503582c

[118] X. Zhou , Q. Zhang , L. Gan , H. Li , T. Zhai , Adv. Funct. Mater. 2016, DOI: 10.1002/adfm.201600318.

[119] C. Zhang , H. Yin , M. Han , Z. Dai , H. Pang , Y. Zheng , Y. Q. Lan , J. Bao , J. Zhu , ACS Nano 2014, 8, 3761. 24601530 10.1021/nn5004315

[120] P. Yu , X. Yu , W. Lu , H. Lin , L. Sun , K. Du , F. Liu , W. Fu , Q. Zeng , Z. Shen , C. Jin , Q. J. Wang , Z. Liu , Adv. Funct. Mater. 2016, 26,137.

[121] T. Pei , L. Bao , G. Wang , R. Ma , H. Yang , J. Li , C. Gu , S. Pantelides , S. Du , H.‐J. Gao , Appl. Phys. Lett. 2016, 108, 053506.

[122] Y. Huang , K. Xu , Z. Wang , T. A. Shifa , Q. Wang , F. Wang , C. Jiang , J. He , Nanoscale 2015, 7, 17375. 26426304 10.1039/c5nr05989e

[123] S. I. Kim , S. Hwang , S. Y. Kim , W. J. Lee , D. W. Jung , K. S. Moon , H. J. Park , Y. J. Cho , Y. H. Cho , J. H. Kim , D. J. Yun , K. H. Lee , I. T. Han , K. Lee , Y. Sohn , Sci. Rep. 2016, 6, 19733. 26792630 10.1038/srep19733 PMC4726434

[124] D. G. Mead , J. C. Irwin , Solid State Commun. 1976, 20, 885.

[125] Y. Ma , Sci. Bull. 2015, 60, 1789.

[126] P. Sinsermsuksakul , J. Heo , W. Noh , A. S. Hock , R. G. Gordon , Adv. Energy Mater. 2011, 1, 1116.

[127] G. Radovsky , R. Popovitz‐Biro , M. Staiger , K. Gartsman , C. Thomsen , T. Lorenz , G. Seifert , R. Tenne , Angew. Chem. Int. Ed. 2011, 50, 12316. 10.1002/anie.201104520 22038979

[128] L. A. Burton , D. Colombara , R. D. Abellon , F. C. Grozema , L. M. Peter , T. J. Savenije , G. Dennler , A. Walsh , Chem. Mater. 2013, 25, 4908.

[129] X. Liu , Y. Li , B. Zhou , X. Wang , A. N. Cartwright , M. T. Swihart , Chem. Mater. 2014, 26, 3515.

[130] J. H. Ahn , M. J. Lee , H. Heo , J. H. Sung , K. Kim , H. Hwang , M. H. Jo , Nano Lett. 2015, 15, 3703. 25933199 10.1021/acs.nanolett.5b00079

[131] A. C. Ferrari , J. C. Meyer , V. Scardaci , C. Casiraghi , M. Lazzeri , F. Mauri , S. Piscanec , D. Jiang , K. S. Novoselov , S. Roth , A. K. Geim , Phys. Rev. Lett. 2006, 97, 187401. 17155573 10.1103/PhysRevLett.97.187401

[132] A. J. Smith , P. E. Meek , W. Y. Liang , J. Phys. C: Solid State Phys. 1977, 10, 1321.

[133] M. Y. Han , B. Ozyilmaz , Y. Zhang , P. Kim , Phys. Rev. Lett. 2007, 98, 206805. 17677729 10.1103/PhysRevLett.98.206805

[134] F. Xia , T. Mueller , Y. M. Lin , A. Valdes‐Garcia , P. Avouris , Nat. Nanotechnol. 2009, 4, 839. 19893532 10.1038/nnano.2009.292

[135] C. H. Liu , Y. C. Chang , T. B. Norris , Z. Zhong , Nat. Nanotechnol. 2014, 9, 273. 24633521 10.1038/nnano.2014.31

[136] E. J. Lee , K. Balasubramanian , R. T. Weitz , M. Burghard , K. Kern , Nat. Nanotechnol. 2008, 3, 486. 18685636 10.1038/nnano.2008.172

[137] F. H. Koppens , T. Mueller , P. Avouris , A. C. Ferrari , M. S. Vitiello , M. Polini , Nat. Nanotechnol. 2014, 9, 780. 25286273 10.1038/nnano.2014.215

[138] A. Rahman , J. W. Guikema , N. Markovic , Nano Lett. 2014, 14, 6621. 25343536 10.1021/nl503276s

[139] S. Jang , E. Hwang , Y. Lee , S. Lee , J. H. Cho , Nano Lett. 2015, 15, 2542. 25811444 10.1021/acs.nanolett.5b00105

[140] J. Z. Ou , W. Ge , B. Carey , T. Daeneke , A. Rotbart , W. Shan , Y. Wang , Z. Fu , A. F. Chrimes , W. Wlodarski , S. P. Russo , Y. X. Li , K. Kalantar‐Zadeh , ACS Nano 2015, 9, 10313. 26447741 10.1021/acsnano.5b04343

[141] W. Sun , X. Rui , D. Yang , Z. Sun , B. Li , W. Zhang , Y. Zong , S. Madhavi , S. Dou , Q. Yan , ACS Nano 2015, 9, 11371. 26487194 10.1021/acsnano.5b05229

[142] Y. Huang , H. X. Deng , K. Xu , Z. X. Wang , Q. S. Wang , F. M. Wang , F. Wang , X. Y. Zhan , S. S. Li , J. W. Luo , J. He , Nanoscale 2015, 7, 14093. 26243183 10.1039/c5nr04174k

[143] G. Ham , S. Shin , J. Park , H. Choi , J. Kim , Y. A. Lee , H. Seo , H. Jeon , ACS Appl. Mater. Interfaces 2013, 5, 8889. 24041133 10.1021/am401127s

[144] T. Rath , L. Gury , I. Sanchez‐Molina , L. Martinez , S. A. Haque , Chem. Commun. 2015, 51, 10198. 10.1039/c5cc03125g PMC4479932 26016404

[145] S. Li , J. Zheng , S. Zuo , Z. Wu , P. Yan , F. Pan , RSC Adv. 2015, 5, 46941.

[146] Y. Tao , X. Wu , W. Wang , J. Wang , J. Mater. Chem. C 2015, 3,1347.

[147] J.‐J. Wu , Y.‐R. Tao , Y. Wu , X.‐C. Wu , Sens. Actuators, B 2016, 231, 211.

[148] P. A. Fernandes , M. G. Sousa , P. M. P. Salomé , J. P. Leitão , A. F. da Cunha , CrystEngComm 2013, 15, 10278.

[149] J. Xia , D. Zhu , L. Wang , B. Huang , X. Huang , X.‐M. Meng ,Adv. Funct. Mater. 2015, 25, 4255.

[150] X. Ling , S. Huang , E. H. Hasdeo , L. Liang , W. M. Parkin , Y. Tatsumi , A. R. Nugraha , A. A. Puretzky , P. M. Das , B. G. Sumpter , D. B. Geohegan , J. Kong , R. Saito , M. Drndic , V. Meunier , M. S. Dresselhaus , Nano Lett. 2016, 16, 2260. 26963685 10.1021/acs.nanolett.5b04540

[151] E. Lorchat , G. Froehlicher , S. Berciaud , ACS Nano 2016, 10,2752. 26820232 10.1021/acsnano.5b07844

[152] M. Hafeez , L. Gan , H. Li , Y. Ma , T. Zhai , Adv. Funct. Mater. 2016, DOI: 10.1002/adfm.201601019. 10.1002/adma.201601977 27391694

[153] D. A. Chenet , O. B. Aslan , P. Y. Huang , C. Fan , A. M. van der Zande , T. F. Heinz , J. C. Hone , Nano Lett. 2015, 15, 5667. 26280493 10.1021/acs.nanolett.5b00910

[154] R. He , J. A. Yan , Z. Yin , Z. Ye , G. Ye , J. Cheng , J. Li , C. H. Lui ,Nano Lett. 2016, 16, 1404. 26757027 10.1021/acs.nanolett.5b04925

[155] L. Lin , J. Li , H. Ren , A. L. Koh , N. Kang , H. Peng , H. Q. Xu , Z. Liu , ACS Nano 2016, 10, 2922. 26832229 10.1021/acsnano.6b00041

[156] D. Geng , L. Meng , B. Chen , E. Gao , W. Yan , H. Yan , B. Luo , J. Xu , H. Wang , Z. Mao , Z. Xu , L. He , Z. Zhang , L. Peng , G. Yu ,Adv. Mater. 2014, 26, 6423. 25043403 10.1002/adma.201401277

[157] C. Y. Su , A. Y. Lu , C. Y. Wu , Y. T. Li , K. K. Liu , W. Zhang , S. Y. Lin , Z. Y. Juang , Y. L. Zhong , F. R. Chen , L. J. Li , Nano Lett. 2011, 11, 3612. 21834558 10.1021/nl201362n

[158] T. Wu , X. Zhang , Q. Yuan , J. Xue , G. Lu , Z. Liu , H. Wang , F. Ding , Q. Yu , X. Xie , M. Jiang , Nat. Mater. 2016, 15, 43. 26595118 10.1038/nmat4477

[159] S. Bae , H. Kim , Y. Lee , X. Xu , J. S. Park , Y. Zheng , J. Balakrishnan , T. Lei , H. R. Kim , Y. I. Song , Y. J. Kim , K. S. Kim , B. Ozyilmaz , J. H. Ahn , B. H. Hong , S. Iijima , Nat. Nanotechnol. 2010, 5,574. 20562870 10.1038/nnano.2010.132

[160] A. Mohsin , L. Liu , P. Liu , W. Deng , I. N. Ivanov , G. Li , O. E. Dyck , G. Duscher , J. R. Dunlap , K. Xiao , G. Gu , ACS Nano 2013, 7, 8924. 24004046 10.1021/nn4034019

[161] I. Bilgin , F. Liu , A. Vargas , A. Winchester , M. K. Man , M. Upmanyu , K. M. Dani , G. Gupta , S. Talapatra , A. D. Mohite , S. Kar , ACS Nano 2015, 9, 8822. 26256639 10.1021/acsnano.5b02019

[162] D. Dumcenco , D. Ovchinnikov , K. Marinov , P. Lazic , M. Gibertini , N. Marzari , O. L. Sanchez , Y. C. Kung , D. Krasnozhon , M. W. Chen , S. Bertolazzi , P. Gillet , I. M. A. Fontcuberta , A. Radenovic , A. Kis , ACS Nano 2015, 9, 4611. 25843548 10.1021/acsnano.5b01281 PMC4415455

[163] A. Govind Rajan , J. H. Warner , D. Blankschtein , M. S. Strano ,ACS Nano 2016, 10, 4330. 26937889 10.1021/acsnano.5b07916

[164] A. Gurarslan , Y. Yu , L. Su , F. Suarez , S. Yao , Y. Zhu , M. Ozturk , Y. Zhang , L. Cao , ACS Nano 2014, 8, 11522. 25347296 10.1021/nn5057673

[165] B. Li , L. Huang , M. Zhong , N. Huo , Y. Li , S. Yang , C. Fan , J. Yang , W. Hu , Z. Wei , J. Li , ACS Nano 2015, 9, 1257. 25584859 10.1021/nn505048y

[166] P. Waduge , I. Bilgin , J. Larkin , R. Y. Henley , K. Goodfellow , A. C. Graham , D. C. Bell , N. Vamivakas , S. Kar , M. Wanunu ,ACS Nano 2015, 9, 7352. 26111109 10.1021/acsnano.5b02369 PMC5142633

[167] Q. Feng , Y. Zhu , J. Hong , M. Zhang , W. Duan , N. Mao , J. Wu , H. Xu , F. Dong , F. Lin , C. Jin , C. Wang , J. Zhang , L. Xie , Adv. Mater. 2014, 26, 2648. 24677312 10.1002/adma.201306095

[168] Y. H. Lee , X. Q. Zhang , W. Zhang , M. T. Chang , C. T. Lin , K. D. Chang , Y. C. Yu , J. T. Wang , C. S. Chang , L. J. Li , T. W. Lin , Adv. Mater. 2012, 24, 2320. 22467187 10.1002/adma.201104798

[169] C. Ahn , J. Lee , H. U. Kim , H. Bark , M. Jeon , G. H. Ryu , Z. Lee , G. Y. Yeom , K. Kim , J. Jung , Y. Kim , C. Lee , T. Kim , Adv. Mater. 2015, 27, 5223. 26257314 10.1002/adma.201501678

[170] W. Chen , J. Zhao , J. Zhang , L. Gu , Z. Yang , X. Li , H. Yu , X. Zhu , R. Yang , D. Shi , X. Lin , J. Guo , X. Bai , G. Zhang , J. Am. Chem. Soc. 2015, 137, 15632. 26623946 10.1021/jacs.5b10519

[171] X. Wang , H. Feng , Y. Wu , L. Jiao , J. Am. Chem. Soc. 2013, 135, 5304. 23489053 10.1021/ja4013485

[172] K. K. Liu , W. Zhang , Y. H. Lee , Y. C. Lin , M. T. Chang , C. Y. Su , C. S. Chang , H. Li , Y. Shi , H. Zhang , C. S. Lai , L. J. Li , Nano Lett. 2012, 12, 1538. 22369470 10.1021/nl2043612

[173] S. Najmaei , Z. Liu , W. Zhou , X. Zou , G. Shi , S. Lei , B. I. Yakobson , J. C. Idrobo , P. M. Ajayan , J. Lou , Nat. Mater. 2013, 12, 754. 23749265 10.1038/nmat3673

[174] A. M. van der Zande , P. Y. Huang , D. A. Chenet , T. C. Berkelbach , Y. You , G. H. Lee , T. F. Heinz , D. R. Reichman , D. A. Muller , J. C. Hone , Nat. Mater. 2013, 12, 554. 23644523 10.1038/nmat3633

[175] Y. Zhan , Z. Liu , S. Najmaei , P. M. Ajayan , J. Lou , Small 2012, 8, 966. 22334392 10.1002/smll.201102654

[176] Y. H. Chang , W. Zhang , Y. Zhu , Y. Han , J. Pu , J. K. Chang , W. T. Hsu , J. K. Huang , C. L. Hsu , M. H. Chiu , T. Takenobu , H. Li , C. I. Wu , W. H. Chang , A. T. Wee , L. J. Li , ACS Nano 2014, 8,8582. 25094022 10.1021/nn503287m

[177] G. W. Shim , K. Yoo , S. B. Seo , J. Shin , D. Y. Jung , I. S. Kang , C. W. Ahn , B. J. Cho , S. Y. Choi , ACS Nano 2014, 8, 6655. 24987802 10.1021/nn405685j

[178] Y. Gong , G. Ye , S. Lei , G. Shi , Y. He , J. Lin , X. Zhang , R. Vajtai , S. T. Pantelides , W. Zhou , B. Li , P. M. Ajayan , Adv. Funct. Mater. 2016, 26, 2009.

[179] J. S. Rhyee , J. Kwon , P. Dak , J. H. Kim , S. M. Kim , J. Park , Y. K. Hong , W. G. Song , I. Omkaram , M. A. Alam , S. Kim ,Adv. Mater. 2016, 28, 2316. 26755196 10.1002/adma.201504789

[180] X. Lu , M. I. Utama , J. Lin , X. Gong , J. Zhang , Y. Zhao , S. T. Pantelides , J. Wang , Z. Dong , Z. Liu , W. Zhou , Q. Xiong , Nano Lett. 2014, 14, 2419. 24678857 10.1021/nl5000906

[181] J. C. Shaw , H. Zhou , Y. Chen , N. O. Weiss , Y. Liu , Y. Huang , X. Duan , Nano Res. 2015, 7, 511.

[182] Z. Q. Xu , Y. Zhang , S. Lin , C. Zheng , Y. L. Zhong , X. Xia , Z. Li , P. J. Sophia , M. S. Fuhrer , Y. B. Cheng , Q. Bao , ACS Nano 2015, 9, 6178. 25961515 10.1021/acsnano.5b01480

[183] S. M. Eichfeld , L. Hossain , Y. C. Lin , A. F. Piasecki , B. Kupp , A. G. Birdwell , R. A. Burke , N. Lu , X. Peng , J. Li , A. Azcatl , S. McDonnell , R. M. Wallace , M. J. Kim , T. S. Mayer , J. M. Redwing , J. A. Robinson , ACS Nano 2015, 9, 2080. 25625184 10.1021/nn5073286

[184] J. Chen , B. Liu , Y. Liu , W. Tang , C. T. Nai , L. Li , J. Zheng , L. Gao , Y. Zheng , H. S. Shin , H. Y. Jeong , K. P. Loh , Adv. Mater. 2015, 27, 6722. 26414106 10.1002/adma.201503446

[185] H. Zhou , C. Wang , J. C. Shaw , R. Cheng , Y. Chen , X. Huang , Y. Liu , N. O. Weiss , Z. Lin , Y. Huang , X. Duan , Nano Lett. 2015, 15,709. 25434747 10.1021/nl504256y PMC4296926

[186] C. Fan , Y. Li , F. Lu , H.‐X. Deng , Z. Wei , J. Li , RSC Adv. 2016, 6, 422.

[187] R. Schlaf , N. R. Armstrong , B. A. Parkinson , C. Pettenkofer , W. Jaegermann , Surf. Sci. 1997, 385, 1.

[188] L. Huang , Y. Yu , C. Li , L. Cao , J. Phys. Chem. C 2013, 117, 6469.

[189] Q. Fu , L. Yang , W. Wang , A. Han , J. Huang , P. Du , Z. Fan , J. Zhang , B. Xiang , Adv. Mater. 2015, 27, 4732. 26153276 10.1002/adma.201500368

[190] H. Li , X. Duan , X. Wu , X. Zhuang , H. Zhou , Q. Zhang , X. Zhu , W. Hu , P. Ren , P. Guo , L. Ma , X. Fan , X. Wang , J. Xu , A. Pan , J. Am. Chem. Soc. 2014, 136, 3756. 24564365 10.1021/ja500069b

[191] H. Li , Q. Zhang , X. Duan , X. Wu , X. Fan , X. Zhu , X. Zhuang , W. Hu , H. Zhou , A. Pan , J. Am. Chem. Soc. 2015, 137, 5284. 25871953 10.1021/jacs.5b01594

[192] X. Duan , C. Wang , Z. Fan , G. Hao , L. Kou , U. Halim , H. Li , X. Wu , Y. Wang , J. Jiang , A. Pan , Y. Huang , R. Yu , Nano Lett. 2016, 16, 264. 26633760 10.1021/acs.nanolett.5b03662

[193] W. Zhang , X. Li , T. Jiang , J. Song , Y. Lin , L. Zhu , X. Xu , Nanoscale 2015, 7, 13554. 26204564 10.1039/c5nr02515j

[194] S. H. Su , Y. T. Hsu , Y. H. Chang , M. H. Chiu , C. L. Hsu , W. T. Hsu , W. H. Chang , J. H. He , L. J. Li , Small 2014, 10, 2589. 24610642 10.1002/smll.201302893

[195] L. Fu , Phys. Rev. Lett. 2011, 106, 106802. 21469822 10.1103/PhysRevLett.106.106802

[196] P. Dziawa , B. J. Kowalski , K. Dybko , R. Buczko , A. Szczerbakow , M. Szot , E. Lusakowska , T. Balasubramanian , B. M. Wojek , M. H. Berntsen , O. Tjernberg , T. Story , Nat. Mater. 2012, 11, 1023. 23023551 10.1038/nmat3449

[197] P. Perumal , R. K. Ulaganathan , R. Sankar , Y.‐M. Liao , T.‐M. Sun , M.‐W. Chu , F. C. Chou , Y.‐T. Chen , M.‐H. Shih , Y.‐F. Chen ,Adv. Funct. Mater. 2016, 26, 3630.

[198] H. Ago , S. Fukamachi , H. Endo , P. Solis‐Fernandez , R. Mohamad Yunus , Y. Uchida , V. Panchal , O. Kazakova , M. Tsuji , ACS Nano 2016, 10, 3233. 26943750 10.1021/acsnano.5b05879

[199] F. Ceballos , M. Z. Bellus , H. Y. Chiu , H. Zhao , ACS Nano 2014, 8, 12717. 25402669 10.1021/nn505736z

[200] Y. Deng , Z. Luo , N. J. Conrad , H. Liu , Y. Gong , S. Najmaei , P. M. Ajayan , J. Lou , X. Xu , P. D. Ye , ACS Nano 2014, 8,8292. 25019534 10.1021/nn5027388

[201] K. Z. Du , X. Z. Wang , Y. Liu , P. Hu , M. I. Utama , C. K. Gan , Q. Xiong , C. Kloc , ACS Nano 2016, 10, 1738. 26607168 10.1021/acsnano.5b05927

[202] Y. Gong , Z. Lin , G. Ye , G. Shi , S. Feng , Y. Lei , A. L. Elias , N. Perea‐Lopez , R. Vajtai , H. Terrones , Z. Liu , M. Terrones , P. M. Ajayan , ACS Nano 2015, 9, 11658. 26502824 10.1021/acsnano.5b05594

[203] Y. Kobayashi , S. Sasaki , S. Mori , H. Hibino , Z. Liu , K. Watanabe , T. Taniguchi , K. Suenaga , Y. Maniwa , Y. Miyata , ACS Nano 2015, 9, 4056. 25809222 10.1021/acsnano.5b00103

[204] J. A. Miwa , M. Dendzik , S. S. Gronborg , M. Bianchi , J. V. Lauritsen , P. Hofmann , S. Ulstrup , ACS Nano 2015, 9, 6502. 26039108 10.1021/acsnano.5b02345

[205] A. Pezeshki , S. H. Hosseini Shokouh , P. J. Jeon , I. Shackery , J. S. Kim , I. K. Oh , S. C. Jun , H. Kim , S. Im , ACS Nano 2016, 10, 1118. 26631357 10.1021/acsnano.5b06419

[206] L. Fu , Y. Sun , N. Wu , R. G. Mendes , L. Chen , Z. Xu , T. Zhang , M. H. Rummeli , B. Rellinghaus , D. Pohl , L. Zhuang , ACS Nano 2016, 10, 2063. 26756578 10.1021/acsnano.5b06254

[207] H. Qiao , J. Yuan , Z. Xu , C. Chen , S. Lin , Y. Wang , J. Song , Y. Liu , Q. Khan , H. Y. Hoh , C. X. Pan , S. Li , Q. Bao , ACS Nano 2015, 9, 1886. 25598406 10.1021/nn506920z

[208] T. Roy , M. Tosun , X. Cao , H. Fang , D. H. Lien , P. Zhao , Y. Z. Chen , Y. L. Chueh , J. Guo , A. Javey , ACS Nano 2015, 9,2071. 25598307 10.1021/nn507278b

[209] S. Wang , X. Wang , J. H. Warner , ACS Nano 2015, 9, 5246. 25895108 10.1021/acsnano.5b00655

[210] J. M. Woods , Y. Jung , Y. Xie , W. Liu , Y. Liu , H. Wang , J. J. Cha ,ACS Nano 2016, 10, 2004. 26836122 10.1021/acsnano.5b06126

[211] Y. Xue , Y. Zhang , Y. Liu , H. Liu , J. Song , J. Sophia , J. Liu , Z. Xu , Q. Xu , Z. Wang , J. Zheng , S. Li , Q. Bao , ACS Nano 2016, 10,573. 26647019 10.1021/acsnano.5b05596

[212] J. Yuan , S. Najmaei , Z. Zhang , J. Zhang , S. Lei , P. M. Ajayan , B. I. Yakobson , J. Lou , ACS Nano 2015, 9, 555. 25569715 10.1021/nn505809d

[213] K. Zhang , T. Zhang , G. Cheng , T. Li , S. Wang , W. Wei , X. Zhou , W. Yu , Y. Sun , P. Wang , D. Zhang , C. Zeng , X. Wang , W. Hu , H. J. Fan , G. Shen , X. Chen , X. Duan , K. Chang , N. Dai , ACS Nano 2016, 10, 3852. 26950255 10.1021/acsnano.6b00980

[214] L. Zhang , Y. Yan , H. C. Wu , D. Yu , Z. M. Liao , ACS Nano 2016, 10, 3816. 26930548 10.1021/acsnano.6b00659

[215] Y. Lee , J. Kwon , E. Hwang , C. H. Ra , W. J. Yoo , J. H. Ahn , J. H. Park , J. H. Cho , Adv. Mater. 2015, 27, 41. 25327379 10.1002/adma.201402271

[216] P. T. Loan , W. Zhang , C. T. Lin , K. H. Wei , L. J. Li , C. H. Chen ,Adv. Mater. 2014, 26, 4838. 24841824 10.1002/adma.201401084

[217] G. W. Mudd , S. A. Svatek , L. Hague , O. Makarovsky , Z. R. Kudrynskyi , C. J. Mellor , P. H. Beton , L. Eaves , K. S. Novoselov , Z. D. Kovalyuk , E. E. Vdovin , A. J. Marsden , N. R. Wilson , A. Patane , Adv. Mater. 2015, 27, 3760. 25981798 10.1002/adma.201500889 PMC4768130

[218] Y. Gong , S. Lei , G. Ye , B. Li , Y. He , K. Keyshar , X. Zhang , Q. Wang , J. Lou , Z. Liu , R. Vajtai , W. Zhou , P. M. Ajayan , Nano Lett. 2015, 15, 6135. 26237631 10.1021/acs.nanolett.5b02423

[219] D. Jariwala , S. L. Howell , K. S. Chen , J. Kang , V. K. Sangwan , S. A. Filippone , R. Turrisi , T. J. Marks , L. J. Lauhon , M. C. Hersam , Nano Lett. 2016, 16, 497. 26651229 10.1021/acs.nanolett.5b04141

[220] S. Rathi , I. Lee , D. Lim , J. Wang , Y. Ochiai , N. Aoki , K. Watanabe , T. Taniguchi , G. H. Lee , Y. J. Yu , P. Kim , G. H. Kim , Nano Lett. 2015, 15, 5017. 26091357 10.1021/acs.nanolett.5b01030

[221] S. Tongay , W. Fan , J. Kang , J. Park , U. Koldemir , J. Suh , D. S. Narang , K. Liu , J. Ji , J. Li , R. Sinclair , J. Wu , Nano Lett. 2014, 14, 3185. 24845201 10.1021/nl500515q

[222] F. Wang , Z. Wang , K. Xu , Q. Wang , Y. Huang , L. Yin , J. He , Nano Lett. 2015, 15, 7558. 26469092 10.1021/acs.nanolett.5b03291

[223] J. H. Yu , H. R. Lee , S. S. Hong , D. Kong , H. W. Lee , H. Wang , F. Xiong , S. Wang , Y. Cui , Nano Lett. 2015, 15, 1031. 25590995 10.1021/nl503897h

[224] X. Q. Zhang , C. H. Lin , Y. W. Tseng , K. H. Huang , Y. H. Lee , Nano Lett. 2015, 15, 410. 25494614 10.1021/nl503744f

[225] X. Wang , L. Huang , Y. Peng , N. Huo , K. Wu , C. Xia , Z. Wei , S. Tongay , J. Li , Nano Res. 2015, 9, 507.

[226] J. Meng , H. D. Song , C. Z. Li , Y. Jin , L. Tang , D. Liu , Z. M. Liao , F. Xiu , D. P. Yu , Nanoscale 2015, 7, 11611. 26090791 10.1039/c5nr02552d

[227] M. H. Chiu , C. Zhang , H. W. Shiu , C. P. Chuu , C. H. Chen , C. Y. Chang , M. Y. Chou , C. K. Shih , L. J. Li , Nat. Commun. 2015, 6, 7666. 26179885 10.1038/ncomms8666 PMC4518320

[228] Y. C. Lin , R. K. Ghosh , R. Addou , N. Lu , S. M. Eichfeld , H. Zhu , M. Y. Li , X. Peng , M. J. Kim , L. J. Li , R. M. Wallace , S. Datta , J. A. Robinson , Nat. Commun. 2015, 6, 7311. 26088295 10.1038/ncomms8311 PMC4557306

[229] P. Rivera , J. R. Schaibley , A. M. Jones , J. S. Ross , S. Wu , G. Aivazian , P. Klement , K. Seyler , G. Clark , N. J. Ghimire , J. Yan , D. G. Mandrus , W. Yao , X. Xu , Nat. Commun. 2015, 6,6242. 25708612 10.1038/ncomms7242

[230] C. Zhang , Y. Chen , J. K. Huang , X. Wu , L. J. Li , W. Yao , J. Tersoff , C. K. Shih , Nat. Commun. 2016, 6, 10349. 26778119 10.1038/ncomms10349 PMC4735610

[231] Y. Gong , J. Lin , X. Wang , G. Shi , S. Lei , Z. Lin , X. Zou , G. Ye , R. Vajtai , B. I. Yakobson , H. Terrones , M. Terrones , B. K. Tay , J. Lou , S. T. Pantelides , Z. Liu , W. Zhou , P. M. Ajayan , Nat. Mater. 2014, 13, 1135. 25262094 10.1038/nmat4091

[232] C. Huang , S. Wu , A. M. Sanchez , J. J. Peters , R. Beanland , J. S. Ross , P. Rivera , W. Yao , D. H. Cobden , X. Xu , Nat. Mater. 2014, 13, 1096. 25150560 10.1038/nmat4064

[233] X. Duan , C. Wang , J. C. Shaw , R. Cheng , Y. Chen , H. Li , X. Wu , Y. Tang , Q. Zhang , A. Pan , J. Jiang , R. Yu , Y. Huang , Nat. Nanotechnol. 2014, 9, 1024. 25262331 10.1038/nnano.2014.222 PMC12049235

[234] X. Hong , J. Kim , S. F. Shi , Y. Zhang , C. Jin , Y. Sun , S. Tongay , J. Wu , F. Wang , Nat. Nanotechnol. 2014, 9, 682. 25150718 10.1038/nnano.2014.167

[235] M. Massicotte , P. Schmidt , F. Vialla , K. G. Schadler , A. Reserbat‐Plantey , K. Watanabe , T. Taniguchi , K. J. Tielrooij , F. H. Koppens , Nat. Nanotechnol. 2016, 11, 42. 26436565 10.1038/nnano.2015.227

[236] S. F. Shi , F. Wang , Nat. Nanotechnol. 2014, 9, 664. 25182037 10.1038/nnano.2014.186

[237] W. J. Yu , Y. Liu , H. Zhou , A. Yin , Z. Li , Y. Huang , X. Duan , Nat. Nanotechnol. 2013, 8, 952. 24162001 10.1038/nnano.2013.219 PMC4249654

[238] H. Yuan , X. Liu , F. Afshinmanesh , W. Li , G. Xu , J. Sun , B. Lian , A. G. Curto , G. Ye , Y. Hikita , Z. Shen , S. C. Zhang , X. Chen , M. Brongersma , H. Y. Hwang , Y. Cui , Nat. Nanotechnol. 2015, 10, 707. 26030655 10.1038/nnano.2015.112

[239] M. Eschbach , E. Młyńczak , J. Kellner , J. Kampmeier , M. Lanius , E. Neumann , C. Weyrich , M. Gehlmann , P. Gospodaricˇ , S. Döring , G. Mussler , N. Demarina , M. Luysberg , G. Bihlmayer , T. Schäpers , L. Plucinski , S. Blügel , M. Morgenstern , C. M. Schneider , D. Grützmacher , Nat. Commun. 2015, 6, 8816. 26572278 10.1038/ncomms9816 PMC4660041

[240] M. Y. Li , Y. Shi , C. C. Cheng , L. S. Lu , Y. C. Lin , H. L. Tang , M. L. Tsai , C. W. Chu , K. H. Wei , J. H. He , W. H. Chang , K. Suenaga , L. J. Li , Science 2015, 349, 524. 26228146 10.1126/science.aab4097

[241] N. Huo , J. Yang , L. Huang , Z. Wei , S. S. Li , S. H. Wei , J. Li , Small 2015, 11, 5430. 26296851 10.1002/smll.201501206

[242] J. Xia , D. Zhu , X. Li , L. Wang , L. Tian , J. Li , J. Wang , X. Huang , X.‐M. Meng , Adv. Funct. Mater. 2016, DOI: 10.1002/adfm.201600699.

[243] R. Yan , S. Fathipour , Y. Han , B. Song , S. Xiao , M. Li , N. Ma , V. Protasenko , D. A. Muller , D. Jena , H. G. Xing , Nano Lett. 2015, 15, 5791. 26226296 10.1021/acs.nanolett.5b01792

[244] T. Roy , M. Tosun , M. Hettick , G. H. Ahn , C. Hu , A. Javey , Appl. Phys. Lett. 2016, 108, 083111.

[245] X. Zhang , F. Meng , J. R. Christianson , C. Arroyo‐Torres , M. A. Lukowski , D. Liang , J. R. Schmidt , S. Jin , Nano Lett. 2014, 14, 3047. 24798138 10.1021/nl501000k

[246] R. Browning , P. Plachinda , P. Padigi , R. Solanki , S. Rouvimov , Nanoscale 2016, 8, 2143. 26726993 10.1039/c5nr08006a

[247] C. Gong , C. Huang , J. Miller , L. Cheng , Y. Hao , D. Cobden , J. Kim , R. S. Ruoff , R. M. Wallace , K. Cho , X. Xu , Y. J. Chabal , ACS Nano 2013, 7, 11350. 24219632 10.1021/nn4052138

[248] S. L. Li , K. Komatsu , S. Nakaharai , Y. F. Lin , M. Yamamoto , X. Duan , K. Tsukagoshi , ACS Nano 2014, 8, 12836. 25470503 10.1021/nn506138y

[249] H. Liu , A. T. Neal , P. D. Ye , ACS Nano 2012, 6, 8563. 22957650 10.1021/nn303513c

[250] Z. Li , R. Ye , R. Feng , Y. Kang , X. Zhu , J. M. Tour , Z. Fang ,Adv. Mater. 2015, 27, 5235. 26255655 10.1002/adma.201501888

[251] D. H. Kang , J. Shim , S. K. Jang , J. Jeon , M. H. Jeon , G. Y. Yeom , W. S. Jung , Y. H. Jang , S. Lee , J. H. Park , ACS Nano 2015, 9,1099. 25629805 10.1021/nn5074435

[252] A. Nipane , D. Karmakar , N. Kaushik , S. Karande , S. Lodha ,ACS Nano 2016, 10, 2128. 26789206 10.1021/acsnano.5b06529

[253] P. Zhao , D. Kiriya , A. Azcatl , C. Zhang , M. Tosun , Y. S. Liu , M. Hettick , J. S. Kang , S. McDonnell , K. C. Santosh , J. Guo , K. Cho , R. M. Wallace , A. Javey , ACS Nano 2014, 8, 10808. 25229426 10.1021/nn5047844

[254] X. Liu , D. Qu , J. Ryu , F. Ahmed , Z. Yang , D. Lee , W. J. Yoo ,Adv. Mater. 2016, 28, 2345. 26808483 10.1002/adma.201505154

[255] W. Liu , D. Sarkar , J. Kang , W. Cao , K. Banerjee , ACS Nano 2015, 9, 7904. 26039221 10.1021/nn506512j

[256] H. S. Lee , S. S. Baik , K. Lee , S. W. Min , P. J. Jeon , J. S. Kim , K. Choi , H. J. Choi , J. H. Kim , S. Im , ACS Nano 2015, 9, 8312. 26169189 10.1021/acsnano.5b02785

[257] S. Jo , D. Costanzo , H. Berger , A. F. Morpurgo , Nano Lett. 2015, 15, 1197. 25607653 10.1021/nl504314c

[258] S. Kim , A. Konar , W. S. Hwang , J. H. Lee , J. Lee , J. Yang , C. Jung , H. Kim , J. B. Yoo , J. Y. Choi , Y. W. Jin , S. Y. Lee , D. Jena , W. Choi , K. Kim , Nat. Commun. 2012, 3, 1011. 22910357 10.1038/ncomms2018

[259] J. Chao , Z. Wang , X. Xu , Q. Xiang , W. Song , G. Chen , J. Hu , D. Chen , RSC Adv. 2013, 3, 2746.

[260] Z. V. Popović , M. Holtz , K. Reimann , K. Syassen , Phys. Status Solidi B 1996, 198, 533.

